# Understanding Azole Removal: Contribution of Biodegradation and Bioadsorption

**DOI:** 10.1007/s12010-026-05741-7

**Published:** 2026-05-22

**Authors:** Julia Płatkiewicz, Robert Frankowski, Tomasz Grześkowiak, Alina Pruss, Agnieszka Zgoła-Grześkowiak

**Affiliations:** 1 Institute of Chemistry and Technical Electrochemistry, Poznan University of Technology, Berdychowo 4, Poznań, 60-965 Poland; 2 Institute of Environmental Engineering and Building Installations, Poznan University of Technology, Berdychowo 4, Poznań, 60-965 Poland

**Keywords:** Azoles, Biodegradation, Bioadsorption, Biodegradation products toxicity, Metabolic activity

## Abstract

Conventional biological treatment in wastewater treatment plants (WWTPs) have demonstrated limited efficiency in removing contaminants of emerging concern. This study investigated the biodegradation of nine azole antifungals using activated sludge from two WWTPs. Nearly complete degradation was observed for ketoconazole (within 17 days) and climbazole (28 days). Clotrimazole and epoxiconazole exhibited notable differences in removal efficiency between two sludges, ranging from 45% to 76% and 50% to 87%, respectively. Moderate removal (39–66%) was observed for tebuconazole, flutriafol, and tiabendazole, while the lowest degradation occurred for imazalil (~ 30%) and fluconazole (~ 10%). Sorption studies revealed that bioadsorption played a major role in the removal of tiabendazole and clotrimazole with 37–40% and 33–38% of the initial amount of compounds adsorbed on activated sludge, respectively. A reduction in microbial metabolic activity and total organic carbon after one week is likely related to substrate depletion typical of batch experiments rather than loss of activated sludge functionality. Transformation product analysis confirmed the formation of several metabolites structurally similar to parent compounds, retaining the bioactive azole ring, with similar or slightly lower toxicity compared to initial azoles. These findings highlight the roles of biodegradation and sorption in the removal of azole fungicides in activated sludge systems and indicate the limited capacity of conventional treatment to fully eliminate these compounds and the potential ecological risks related to the presence of azoles and their degradation products in the environment.

**Supplementary Information:**

The online version contains supplementary material available at https://doi.org/10.1007/s12010-026-05741-7.

## Introduction

The continuous identification of chemicals of emerging concern (CECs) and the increasing complexity of contaminant mixtures have raised significant attention toward their occurrence in wastewater systems. These compounds, including pharmaceuticals and personal care products, are commonly detected in domestic, industrial, and agricultural wastewaters, which therefore require effective treatment before discharge into the environment [1–4]. This presents a challenge for wastewater treatment plants (WWTPs), which must manage the removal of diverse types of inorganic and organic pollutants [5].

The conventional wastewater purification begins with preliminary treatment, which aims at removing floating materials and larger suspended solids with the use of screens, grinders, and grit chambers [6]. It is followed by primary treatment, which is based on physical methods such as sedimentation, gravity settling with coagulation and flocculation, as well as flotation, eliminating over 70% of suspended solids and 60% of oil and grease [7, 8]. Secondary treatment is focused mainly on biological methods designed to degrade the organic matter by the microorganisms suspended in a bioreactor or microorganisms attached to inert materials [6, 9].

Among biological methods, the most commonly employed technique is the activated sludge process. It usually consists of three stages: an aeration tank acting as a biological reactor; a sedimentation tank where the separation of treated water and solid activated sludge takes place; and a recycle system to recirculate a part of activated sludge (20% to 50%) to the aeration tank with influent [10]. The remaining part, including the thickened solids, is withdrawn from the treatment process and disposed of as biosolids, which may be used as fertilizer in agriculture [11, 12]. Activated sludge used in WWTPs typically consists of various strains of bacteria, such as *Achromobacter*, *Escherichia coli*, *Flavobacterium*, *Alcaligenes*, *Nocardia*, *Bacillus*, *Pseudomonas*, *Citromonas*, etc. [13], which utilize the organic matter and convert contaminants into simpler, less toxic or non-toxic compounds through microbial metabolism [14]. Biological wastewater treatment methods are widely used due to their affordability, high pollutant removal efficiency, low maintenance requirements, and eco-friendly nature [15].

However, the conventional activated sludge systems are primarily designed for the removal of macro-pollutants, therefore they exhibit limited efficiency in the case of contaminants of emerging concern [16–18]. The removal efficiencies of CECs vary widely among different compound classes such as pharmaceuticals (57.0-76.5% for trimethoprim) [19], UV-filters (30–55% for 2-ethylhexylethoxycinnamate (EHMC)) [20], and biocides (74.5% for the antimicrobial agent triclosan) [21, 22].

Contaminants of emerging concern also include azole compounds, which are characterized by extensive biological activity and are mainly used as antifungal agents. Due to the chemical structure of azoles, the imidazole and triazole derivatives are distinguished. The former class includes substances containing in their molecule a ring with two non-adjacent nitrogen atoms, e.g., climbazole, clotrimazole, ketoconazole, as well as imazalil. They belong to an important group of antifungal agents, characterized by relatively high lipophilicity, and are used in the treatment of fungal infections in humans and animals [23]. Triazole antifungals contain five-membered rings that consist of two carbon atoms and three nitrogen atoms, e.g., fluconazole, tebuconazole, epoxiconazole, and flutriafol. They are frequently used as agricultural fungicides, except for fluconazole, which is used as a human and veterinary drug [24].

Azole antifungals work by blocking ergosterol synthesis through the suppression of the cytochrome P450-dependent 14α-demethylase enzyme encoded by the CYP51 gene [25]. This increases fungal membrane permeability, inhibiting growth and/or causing death [26]. However, azole biocides may adversely affect the endocrine systems of non-target organisms such as *Oncorhynchus mykiss* [27], *Xenopus tropicalis* [28], and *Homo sapiens* [29, 30].

An extensive application of azoles leads to their widespread occurrence in wastewater and environmental waters. For example, climbazole was detected in surface water samples from Germany at concentrations ranging from 47 to 530 ng L^-1^ [31], in China with a maximum concentration of 264 ng L^-1^ [32], but in Poland only up to 19.2 ng L^-1^ [33]. Clotrimazole was found in the United Kingdom (9–27.3 ng L^-1^) [34], in Spain (7.2–9.1 ng L^-1^) [23], and in Poland (up to 187 ng L^-1^) [33], while fluconazole was detected in Switzerland (up to 9 ng L^-1^) [35], in Spain (25–32 ng L^-1^) [23], and in Poland (0.4–300 ng L^-1^) [33]. Ketoconazole was detected in surface waters in South Africa (12.8–34.5 ng L^-1^) [36] and China (up to 1.5 ng L^-1^) [37]. Considerably greater concentrations were found for tebuconazole, for which concentrations in Polish surface water ranged from 0.9 to 1783 ng L^-1^ [33]. Other examples of azole occurrence in the environment can also be found, as pointed out in reviews on the topic [38, 39].

The removal of azole compounds from wastewater has become a serious challenge for wastewater treatment plants, as many rely on conventional methods such as activated sludge and anaerobic-anoxic-oxic techniques. These methods are highly efficient at reducing chemical oxygen demand (COD), biological oxygen demand (BOD), and pathogens. Nonetheless, they are not effective in removing the substances present at very low concentrations (micro or nano-gram per liter) such as azole fungicides [40, 41]. Biodegradation studies using activated sludge conducted by Kahle et al. [35] reported removal efficiencies of 25% for tebuconazole and 40% for clotrimazole after 24 h, while Cai et al. [42] observed 30% removal of fluconazole after 21 days. As a result, WWTPs become a major pathway into the environment for antifungals. The studies indicate that approximately 345 tons of climbazole are utilized annually across China, with an estimated 245 tons potentially entering the environment even after wastewater treatment processes [43]. Therefore, a more comprehensive understanding of the mechanism and fate of azole compounds during the activated sludge process is essential to improve the removal efficiency.

The objective of this research was to investigate the biodegradation of nine azole compounds (ketoconazole, climbazole, tebuconazole, clotrimazole, epoxiconazole, flutriafol, tiabendazole, imazalil, and fluconazole) utilizing activated sludge. Furthermore, the metabolic activity of the microorganisms and the total organic carbon content were assessed. Subsequent to the biodegradation experiments, the activated sludge was also analyzed to verify if bioadsorption of the removed azoles on the activated sludge had occurred. In an attempt to create a degradation pathway, the products of azole compounds degradation were also identified. To the best of the authors’ knowledge, this is the first such extensive study to include tests for the removal of as many as nine azoles under the same conditions, allowing for an in-depth comparison of their behavior in terms of biodegradation and bioadsorption.

## Materials and Methods

### Chemicals and Equipment

The standards of fluconazole (FLU), epoxiconazole (EPO), flutriafol (FLUTR), tiabendazole (TIAB), and imazalil (IMAZ) were purchased from Sigma-Aldrich/Merck (Darmstadt, Germany); climbazole (CLI), ketoconazole (KET), and clotrimazole (CLO) were acquired from Pol-Aura (Poznań, Poland), and tebuconazole (TEB) was obtained from Thermo Fisher Scientific (Waltham, Massachusetts, USA). All were of purity ≥ 98%. The properties of the tested azoles are detailed in Table S1.

The reagents used for the preparation of mineral medium included sodium hydrogen phosphate, potassium dihydrogen phosphate, ammonium chloride, EDTA, iron(II)sulfate heptahydrate, magnesium sulfate heptahydrate, manganese sulfate monohydrate, copper sulphate pentahydrate, zinc sulfate heptahydrate (all with purity ≥ 98%, obtained from Pol-Aura (Poznań, Poland)); cobalt(II) chloride (≥ 98%, Sigma-Aldrich/Merck (Darmstadt, Germany)); calcium chloride dihydrate (99.5%, Chempur (Piekary Śląskie, Poland)) and ammonium heptamolybdate (99.99%, Thermo Fisher Scientific (Waltham, Massachusetts, USA)).

To assess the metabolic activity of bacterial cells, 3-(4,5-dimethylthiazol-2-yl)-2,5-diphenyltetrazolium bromide (MTT), purchased from Sigma Aldrich/Merck (Darmstadt, Germany), and propan-2-ol, supplied by Avantor (Poland), were used. The MS-grade acetonitrile and formic acid (≥ 97.5%), used for the preparation of the chromatographic mobile phase, were both obtained from Merck (Darmstadt, Germany). The ultra-pure water was produced through reverse osmosis using the Demiwa system from Watek (Ledec nad Sazavou, Czech Republic) and subsequently double-distilled in a Heraeus Bi18 quartz apparatus (Hanau, Germany).

### Biodegradation Tests

In order to conduct the biodegradation experiments, activated sludge from two wastewater treatment plants: the WWTP C treating urban sewage (which serves a population equivalent of approximately 1,100,000), and the WWTP B treating rural sewage (which serves a population equivalent of approximately 50,000), was collected and used as inoculum. Both WWTPs use biological treatment technology with enhanced removal of nitrogen and phosphorus compounds (i.e. the biological nutrients removal). The active sludge age in WWTP C varies between 14 and 22 days while the sediment age in WWTP B varies between 25 and 39 days. The Total Suspended Solids for the WWTP C varies between 3190 and 4872 mg L^− 1^, the mineral suspension was from 648 to 996 mg L^− 1^, and the organic suspension from 2542 to 3876 mg L^− 1^. The total suspension for the WWTP B varies between 3644 and 4856 mg L^− 1^, the mineral suspension was from 676 to 827 mg L^− 1^, and the organic suspension from 2938 to 3653 mg L^− 1^. Before the biodegradation test, both activated sludges were subjected to a purification process by two days of aeration.

The biodegradation experiments were conducted based on the OECD (The Organization for Economic Co-operation and Development) 301 guideline [44]. They were carried out in 250 mL glass bottles, separately for each azole compound with an initial concentration of 1 mg L^− 1^. The bottles were filled with 90 mL of mineral medium, 10 mL of activated sludge, and a specific volume of azole compound solution to achieve a concentration of 1 mg L^− 1^. An abiotic control (azoles in medium without activated sludge) was included to evaluate potential non-biological removal pathways. The composition of the mineral medium is presented in Table S2 [45]. Each test was carried out in triplicate. The reactors were placed on an orbital shaker and maintained at room temperature, with access to air and without exposure to light, for the 35 days of the experiment. During this time, samples were withdrawn on days 1, 3, 7, 10, 14, 17, 21, 24, 28, and 35 of the experiment.

### Sample Preparation

Before the LC-MS/MS analysis, the samples from the biodegradation tests were diluted ten times and filtered through PTFE syringe filters with a pore size of 0.2 μm. After finishing the biodegradation process, the contents of the reactors were filtered through a filter paper to investigate whether azole compounds had adsorbed on the activated sludge. The residue was dried, then covered with 20 mL of methanol and extracted on a laboratory orbital shaker for 24 h. The resulting extracts were diluted 10 times, filtered with 0.2 μm PTFE syringe filters, and subjected to the LC-MS/MS analysis.

### LC-MS/MS Analytical Conditions

Azoles were determined using a previously described method [33]. The LC-MS/MS system comprising the UltiMate 3000 HPLC unit (Dionex, Sunnyvale, CA, USA) and the 4000 QTRAP mass spectrometer (ABSciex, Foster City, CA, USA) equipped with a Kinetex Evo C18 column (150 mm x 2.1 mm I.D.; 2.6 μm) from Phenomenex (Torrance, CA, USA) were used. The mobile phase included ultrapure water with 0.1% formic acid (phase A) and acetonitrile (phase B), delivered at a flow rate of 0.3 mL min^− 1^. The sample injection volume was 5 µL, and the column was maintained at 35 °C. The chromatographic separation followed a gradient elution program, increasing and maintaining the organic phase percentage as follows: 0 min − 20%, 4 min − 60%, 4.5 min − 100%, and 6.5 min − 100%.

The column effluent was introduced into the mass spectrometer via an electrospray ionization source (the Turbo Ion Spray) operating in positive ion mode. The mass spectrometer’s operational parameters were: ESI temperature 450 °C, ion spray voltage 4500 V, curtain gas 10 psi, nebulizer gas 40 psi, auxiliary gas 45 psi, collision gas set to high, and a dwell time of 100 ms per mass transition. Detected MRM (Multiple Reaction Monitoring) transitions, along with analyte-specific parameters, are summarized in Table S3 and method validation parameters in Tables S4-S5.

A screening for degradation products was performed in the Enhanced MS mode. The ions selected from the screening were then monitored in the Multiple Reaction Monitoring by selecting the same ions in both Q1 and Q3 quadrupoles. For that purpose a low collision energy of 5 eV was selected. Using the Information Dependent Acquisition mode the ions of relatively high intensity were further fragmented in the Enhanced Product Ion mode. For this purpose collision energy was set to 20 eV with a spread of 5 eV.

### Total Organic Carbon Measurement

Total organic carbon (TOC) was determined using the TOC/TN multi NC 3100 instrument from Analytik Jena (Jena, Germany). A sample volume of 500 µL was used, with the oven maintained at 750 °C. Each sample was injected 3–5 times to ensure a relative standard deviation below 2%. The average values were calculated based on the sample responses and standard curves prepared within a range of 0 to 1000 mg L^− 1^ of carbon.

### Metabolic Activity of Bacteria

The MTT test is based on the enzymatic reduction of yellow, soluble in water, mono-tetrazolium salt, which is composed of a positively charged quaternary tetrazole ring core. The MTT reagent, due to its positive charge and lipophilic nature, can pass through the cell membrane and then is reduced to a violet-blue, water-insoluble formazan molecule by metabolically active cells. Subsequently, the colorimetric analysis of formazan formation enables the assessment of the cell’s metabolic activity [46].

To assess the metabolic activity of bacteria, 900 µL samples were collected from reactors on days 0, 7, 14, 21, 28 and 35 of experiment, and 100 µL of MTT solution at a concentration of 5 mg mL^− 1^ was added. Next, they were mixed by a vortex mixer, placed in the incubator at 37 °C for 120 min, and then subjected to centrifugation. After that, the supernatant was removed, and the sediment was dissolved in 1 mL of propan-2-ol, and mixed for 20 min. The samples were then centrifuged again, and the supernatant was collected for spectrophotometric analysis using a UV-VIS spectrophotometer SPECORD 50 PLUS from Analytik Jena (Jena, Germany). Absorbance measurements were performed at a wavelength of 560 nm.

### Toxicity Evaluation

The toxicity of tested compounds and their degradation products was evaluated using the ECOSAR version 2.0 application developed by the United States Environmental Protection Agency’s Office of Chemical Safety and Pollution Prevention [47]. Based on the chemical structures of the compounds and their similarity to chemicals for which aquatic studies are available, the program evaluates the ecological hazards of untested chemicals. For the purpose of the current study, chronic toxicity (ChV) was assessed, which refers to long-term, low-level exposure effects in ecotoxicology. Calculations were performed at three trophic levels, i.e. to fish, daphnid, and green algae.

### Statistical Analysis

The results were expressed as mean ± standard deviation (for n = three replicates). Comparison of mean values was performed using ANOVA and post-hoc Tukey test. All statistical calculations were done using MS Excel software with Analysis ToolPak.

## Results and Discussion

### Biodegradation of Azoles

**Fig. 1:**
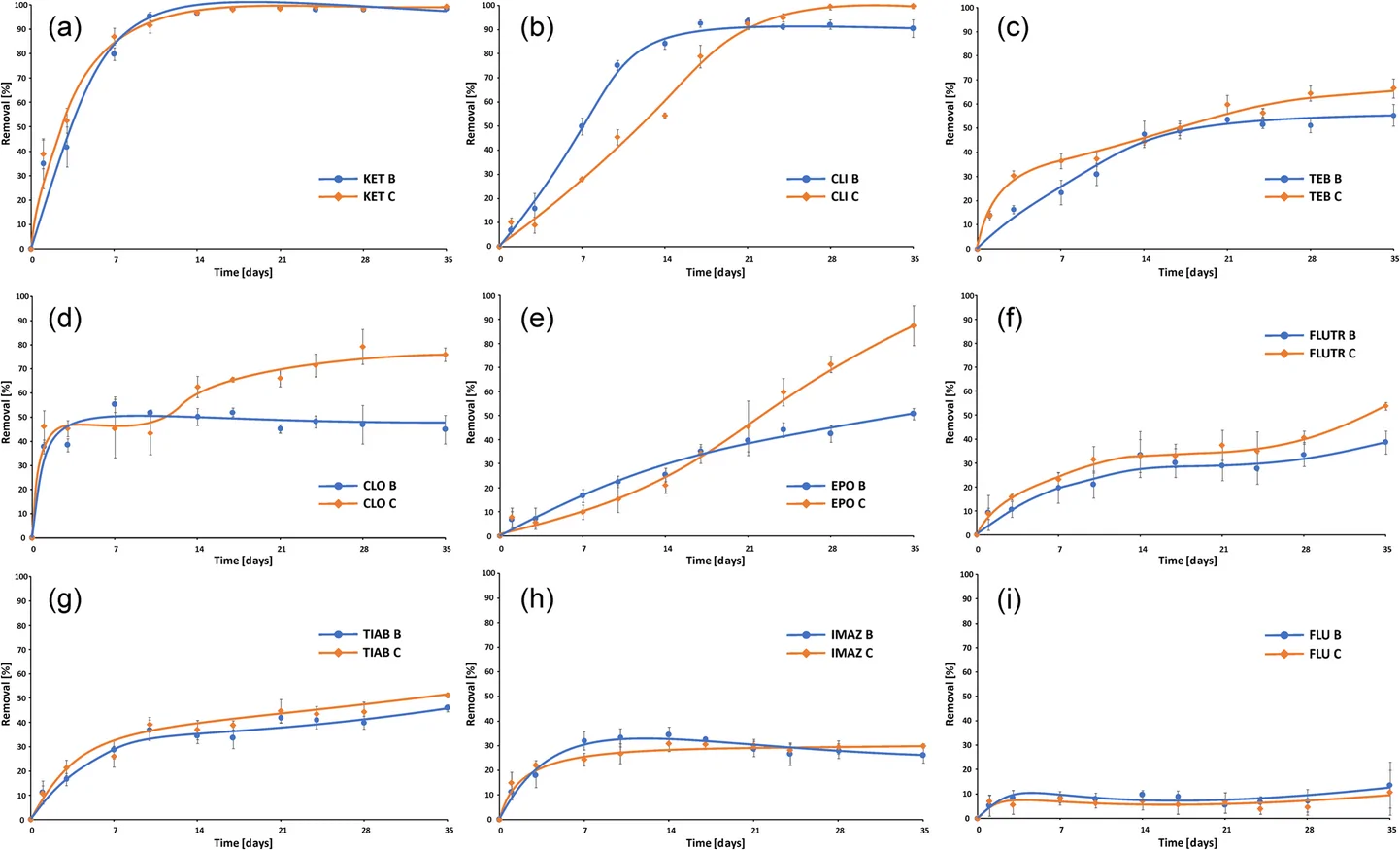
Removal of azoles in the biodegradation experiments with activated sludge from rural WWTP (B) and urban WWTP (C) for (**a**) ketoconazole, (**b**) climbazole, (**c**) tebuconazole, (**d**) clotrimazole, (**e**) epoxiconazole, (**f**) flutriafol, (**g**) tiabendazole, (**h**) imazalil, and (**i**) fluconazole. The bars represent standard deviation

The biodegradation of nine azole compounds in the presence of activated sludge from urban (C) and rural (B) wastewater treatment plants is presented in Fig. 1. The results obtained for the tests with both inocula showed varying degradation depending on the compound studied. Almost complete primary degradation was achieved for KET after 17 days in experiments with both activated sludges, while for CLI, it was reached after 28 days with activated sludge from the urban WWTP. Slightly lower results were obtained for CLI for sludge from the rural WWTP, however, after 17 days, CLI degradation exceeded 90% and then stabilized. In case of TEB, CLO, EPO, FLUTR, and TIAB, the difference between various inocula is clearly visible (and confirmed statistically, Table S6), particularly for CLO with 45% of degradation with activated sludge from WWTP B and 76% of removal with activated sludge from WWTP C, as well as EPO with 50% and 87%, respectively. A similar trend, though with smaller differences, was observed in the biodegradation of TEB, FLUTR, and TIAB, with removal percentages ranging from 39% to 55% in case of WWTP B and from 51% to 66% with WWTP C (Table S6). Lower results of primary degradation after 35 days was obtained for IMAZ with 13% (WWTP B) and 30% (WWTP C). The compound with significantly lower degradation results was fluconazole, reaching only 13% removal using activated sludge from the WWTP B and 10% for sludge from the WWTP C.

**Fig. 2:**
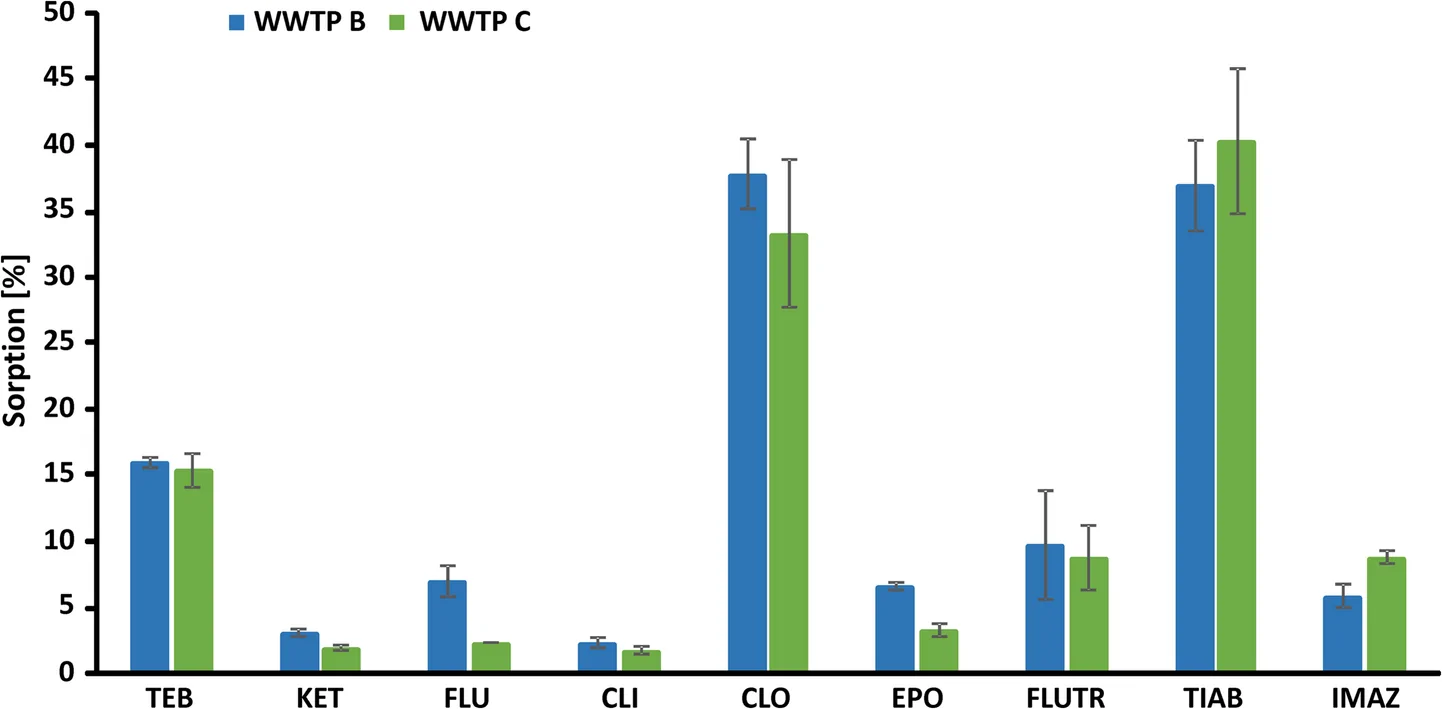
Bioadsorption of tested azole compounds onto the activated sludge from rural WWTP B and urban WWTP C. The bars represent standard deviation

The bar charts in Fig. 2 present the percentage of the initially spiked amount of the azole compound that was adsorbed onto the sludge. Among all tested azoles, the highest values were observed for CLO (38% and 33%) as well as TIAB (37% and 40%) for activated sludge from WWTP B and WWTP C, respectively. Although KET and CLI are characterized by relatively high values of octanol-water partition coefficient (logK_ow_ 4.3 for KET and 3.8 for CLI, Table S1), suggesting a strong potential for bioadsorption, the analysis of their content in the activated sludge at the end of the experiment showed that only a very small percentage was adsorbed (3.0% and 1.8% for KET, 2.2% and 1.6% for CLI, for the sludge from WWTP B and WWTP C, respectively). This confirms that these two compounds were primarily removed through microbial activity.

**Fig. 3:**
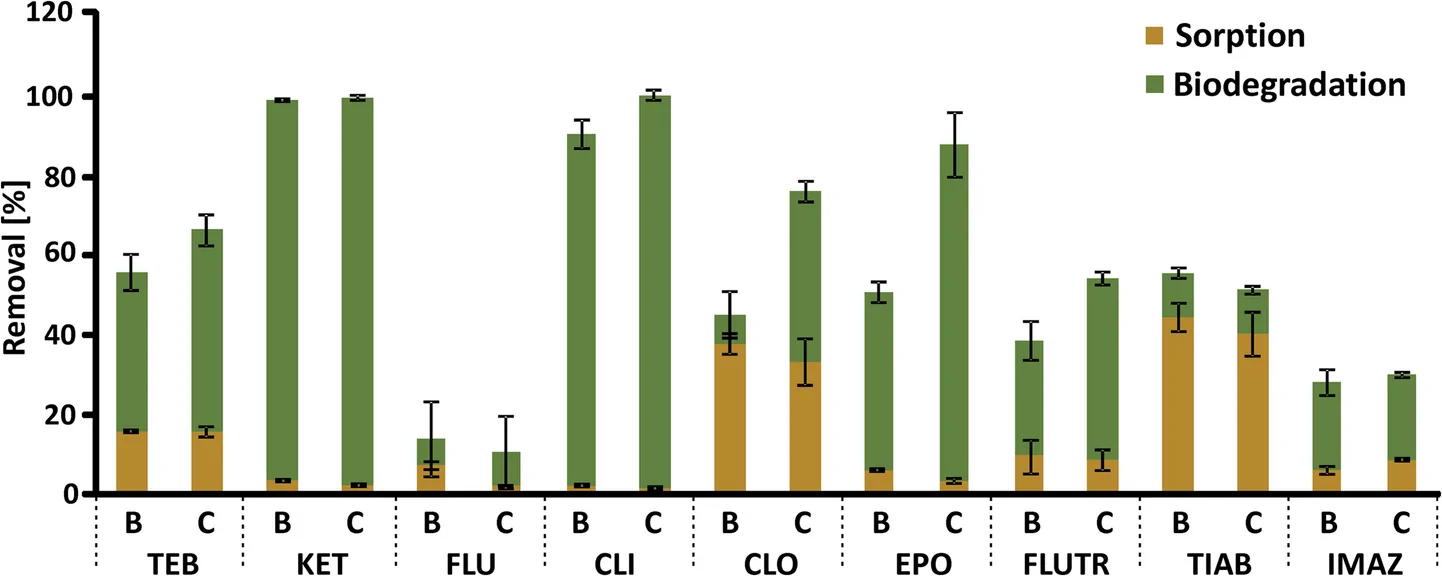
Removal of azoles due to biodegradation and sorption in tests with activated sludge from rural WWTP (B) and with urban WWTP (C). The bars represent standard deviation

A comparison of azole removal by biodegradation and bioadsorption is presented in Fig. 3. The biodegradation share was calculated from the difference between the total removal shown in Fig. 1 and the bioadsorption shown in Fig. 2. It is known, that the activated sludge is a flocculent culture of organisms, and the first step in the biodegradation is diffusion of the compounds from the aqueous phase to the flocs followed by adsorption and degradation. It could be expected that adsorption should be greater for higher log K_ow_. However, log K_ow_ values of KET (4.3), CLI (3.8) and CLO (4.1) are similar while only for CLO adsorption was considerable. This may indicate to faster removal of both KET and CLI, which is confirmed by the data (Fig. 3). On the other hand, the greatest adsorption (and low biodegradation) was observed for both CLO and TIAB, but log K_ow_ of TIAB (2.4) is much lower than that of CLO. This shows that log K_ow_ is not the only one parameter governing adsorption of azoles. As it can be found in Table S1, the low pK_a_ value of TIAB (4.7) makes it unionized in neutral conditions and supports its adsorption, while pK_a_ is greater for CLO (6.12), which makes it partially protonated. As a result, adsorption of both TIAB and CLO can be similar.

The complete degradation of CLI during both experiments reflects the results found in the literature. Cai et al. [42] conducted biodegradation studies with activated sludge reaching the complete removal of CLI after 11 days. The faster degradation compared to the present study is presumably due to the twice lower initial concentration of azoles that may result in reduced microbial toxicity. Nevertheless, other causes should also be considered, such as a different composition of bacterial strains involved in the biodegradation process. On the other hand, complete biodegradation of KET found in the present study contradicts the very low biodegradation reported by Nyirenda et al. [48]. The authors conducted a test using sludge obtained from the university’s domestic WWTP at a concentration of 50 mg L^− 1^ and achieved only 1% biodegradation of KET. Such a significant difference leads us to believe that the main cause may be the origin of the inoculum, which may not have had prior contact with KET and therefore lacks strains adapted to its removal.

Regarding CLO, previous research by Kahle et al. [35] indicated that after 24 h, the biodegradation of CLO with activated sludge stabilized at the level of 40%. However, after the sorption analysis, they stated that this removal is due to the adsorption of CLO on the activated sludge. These findings are consistent with the results obtained in the present study, which showed that although a removal of 45% was observed for WWTP B and 76% for WWTP C, the corresponding bioadsorption percentages were significantly high. This indicates that, particularly in the case of sludge from WWTP B, biodegradation was low, as only about 7% of CLO was actually eliminated by microorganisms. This may be explained by the high hydrophobicity of CLO (logK_ow_ 4.1), and therefore, bioadsorption is preferred. On the other hand, studies conducted by Pacholak et al. [49] on the biodegradation of CLO by bacterial strains (*Aeromonas* PA3, *Alcaligenes faecalis*, and *Aeromonas* AS1) isolated from activated sludge demonstrate that bacteria exhibit moderate degradation ability. Although the initial azole concentration was ten times lower than in the present study, the removal after 36 days was in the range from 45% to 67%, depending on the bacterial strain [49].

The biodegradation of TEB with activated sludge was also conducted by Kahle et al. [35], however, the initial concentration was 100 ng L^− 1^_,_ and the experiment lasted only 24 h. After that time, the removal was around 25% and the sorption on activated sludge was observed, however, lower in comparison with CLO. Zhang et al. [50] performed the biodegradation of TEB and FLUTR in sediment collected from a lake and reported very slow degradation with a half-life (t_1/2_) of 135.88–150.65 days for TEB and 138.60–144.38 days for FLUTR. The EPO removal efficiencies of 51% (WWTP B) and 87% (WWTP C) obtained in the present study indicate the biodegradation potential. It was also confirmed by Alexandrino et al. [14], who successfully studied the removal of EPO by microbial consortia enriched from estuarine sediment and agricultural soil with complete biodegradation of 10 mg L^− 1^ of azole in a maximum of 21 days. Similar results were obtained by Perruchon et al. [51], who investigated the biodegradation of TIAB by a bacterial consortium isolated from soil. The bacteria were able to achieve complete degradation for lower concentrations (75 and 150 mg L^− 1^) in 7 days and a higher concentration (750 mg L^− 1^) in 21 days, showing surprisingly high biodegradability potential. In their research, Papazlatani et al. [52] isolated the fungus *Cladosporium herbarum* from soil contaminated with fungicides and proved its ability to degrade IMAZ at concentrations up to 100 mg L^− 1^, although its performance decreased with higher doses, indicating a detoxification rather than metabolic use. They also tested the removal of IMAZ in a more practical context using a bench-scale bioreactor, achieving the removal of 96% of IMAZ from synthetic wastewater with a concentration of 200 mg L^− 1^, showing the strain potential for application in treatment systems. This result is significantly better than that obtained in our study, despite the fact that we used a significantly lower concentration of IMAZ. Interestingly, although IMAZ is an antifungal agent, in the study by Papazlatani et al. [52] it was readily degraded by *Cladosporium herbarum* fungus.

The low biodegradation of FLU in the present study confirms the results obtained by Cai et al. [42], who stated that 30% of FLU was removed after the first 21 days of the process, and then FLU remained stable for another 56 days. However, the initial concentration of FLU was 500 ng L^− 1^ [42]. Moreover, similar findings were reported by Kahle et al. [35], who observed no degradation of FLU. It is worth noting that the experiment was limited to 24 h, and the initial concentration was only 100 ng L^− 1^. The biodegradation limitations may be due to the presence of strongly electron-withdrawing fluorine atoms in the structure, which can reduce the reactivity of the aromatic ring towards oxidative enzymes like oxygenases and peroxidases, thus lowering its oxidation ability [53]. Low removal of FLU from the aqueous phase was also confirmed by poor bioadsorption on the activated sludge (7% and 2% of initial content on the sludge from WWTP B and C, respectively) due to its particularly low logK_ow_, equal to 0.4, which indicates to FLU’s tendency to remain in the liquid phase, in comparison with other tested azole compounds. Consequently, FLU exhibits low removal efficiency by activated sludge, being especially difficult to eliminate using traditional wastewater treatment technologies.

### Identification of Biodegradation Products and Estimation of Their Toxicity

The results of biodegradation and bioadsorption show that the removal of azoles is problematic. In situations where azoles are removed by biodegradation, it is usually possible to identify their biodegradation products. However, when bioadsorption is the main process involved in the removal of these compounds, as in the case of TIAB and CLO, it is difficult to identify the biodegradation products. It is also obvious that this is impossible when the compound is not removed at all, as is the case with FLU. Nevertheless, for the four most degraded compounds, the degradation products were successfully identified (Figures S1-S13). Toxicity of these azoles and their degradation products was assessed using the ECOSAR application, which estimates toxicity based on chemical structures (Table S7).

**Fig. 4:**
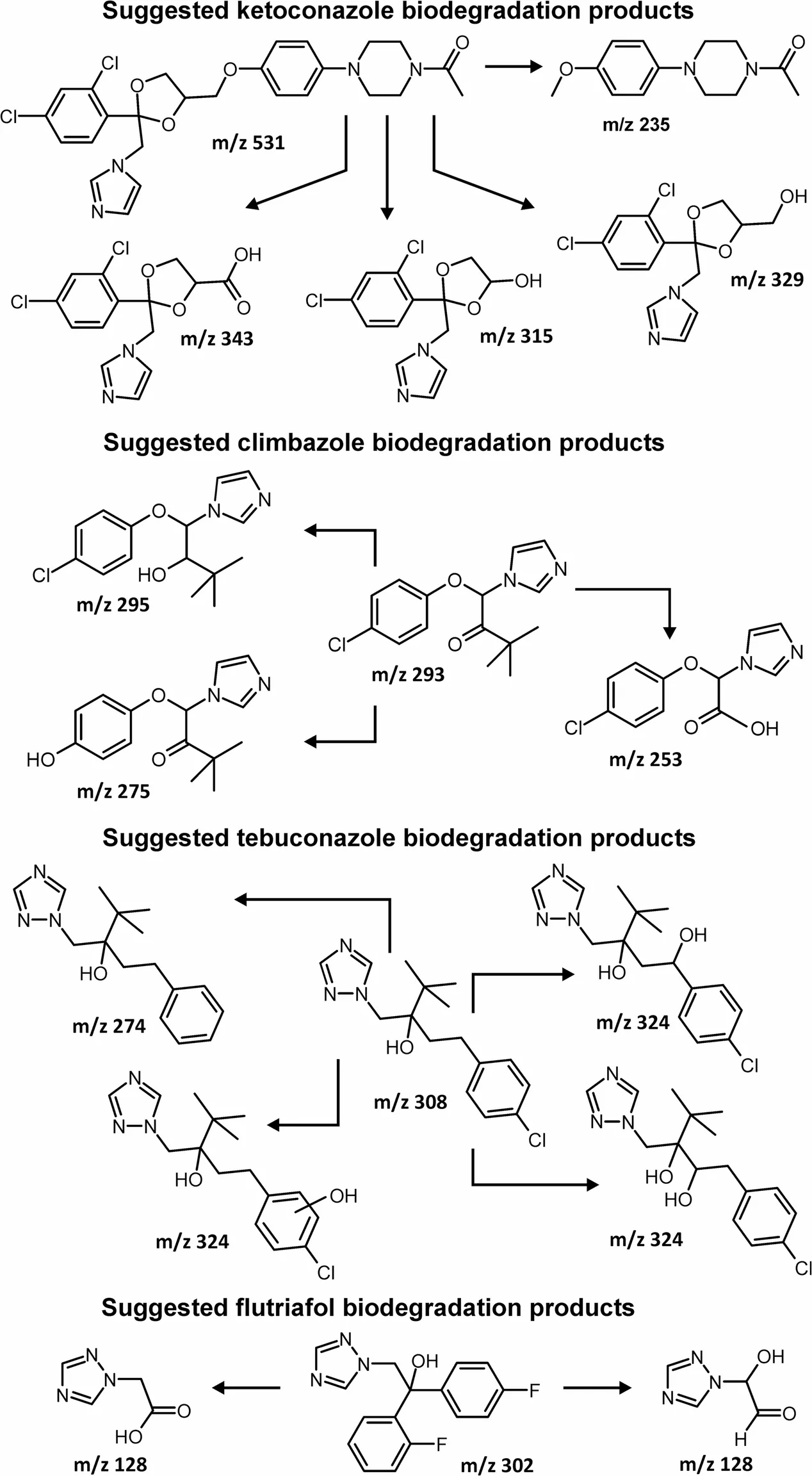
Identified biodegradation products of ketoconazole, climbazole, tebuconazole, and flutriafol

KET and CLI are compounds that have been removed to the highest degree in the tests conducted. Among the biodegradation products of KET, four main compounds were detected at m/z = 343, 329, 315, and 235. All these ions originate from the splitting of the molecule into two parts in the vicinity of the ether group (Fig. 4). None of these products had previously been identified in KET biodegradation tests. Until now, a similar breakdown of the KET molecule has only been reported in catalytic photooxidation studies. However, the reported products, except for one, had a different structure [54]. The toxicity of KET degradation products was slightly lower than that of KET for the products containing the azole ring, while in the case of the product at m/z = 235, it was two orders of magnitude lower for each of the organisms: fish, daphnia, and green algae (Table S7).

Among the biodegradation products of CLI, a few main compounds have been found (Fig. 4). The identified products included those in which changes associated with the degradation of the alkyl chain were observed with protonated molecules recorded at m/z = 295 and 253. On the other hand, a product at m/z = 275 has been detected originating from hydroxylation of the aromatic ring (replacement of the chlorine atom with the hydroxy group). Both these pathways were previously postulated by Cai et al. [42], who tested the biodegradation of CLI under aerobic conditions. Formation of imidazole was also observed (as the ion detected at m/z = 69) as previously found by Pacholak et al. [55]. Interestingly, although the removal of CLI was relatively high, there was only one identified degradation product of considerably lower toxicity (Tabel S6).

TEB and FLUTR are further compounds for which biodegradation products have been identified. The biodegradation products of TEB show two types of intensive ions – at n/z = 324 and 274 (Fig. 4). The ions at m/z = 324 originate from oxidation of both the alkyl chain and aromatic ring and were detected at different retention times, showing multiple positions of the hydroxy group. The ion at m/z = 274 originates from dechlorinated TEB. These biodegradation products were previously reported in other studies on biodegradation of TEB – in wetlands [56] and in soil [57, 58]. Similarly to CLI, TEB biodegradation products were of similar, slightly lower toxicity for each of the organisms: fish, daphnia, and green algae (Table S7).

For FLUTR, only two biodegradation products could be identified (both at m/z = 128), which were not reported previously in biodegradation studies (Fig. 4). However, the biodegradation of FLUTR was incomplete, and adsorption played a significant role in the removal of this compound (and probably of its biodegradation products). It is also possible that the amount of intermediate degradation products at a concentration sufficient for their identification was limited due to their rapid conversion to the two products that we were able to identify. Nevertheless, it is important to note that the identified FLUTR biodegradation products indicate that this compound breaks down into fragments with a structure significantly different from the original compound. What is more important, the toxicity of both identified degradation products is three orders of magnitude lower than that of FLUTR even though both these compounds contain the azole ring in their structures (Table S7).

Analysis of the toxicity of the identified metabolites showed that minor structural changes resulting from biodegradation, in particular the stability of azole rings, lead to the formation of products with similar toxicity to the initial azoles. The results obtained emphasize the important role of complete biodegradation in the removal of azole fungicides in activated sludge systems, while at the same time pointing to the limited ability of conventional treatment methods to completely eliminate all compounds in this group and the potential ecological risks associated with their presence in the environment.

### Metabolic Activity of Bacteria

**Fig. 5:**
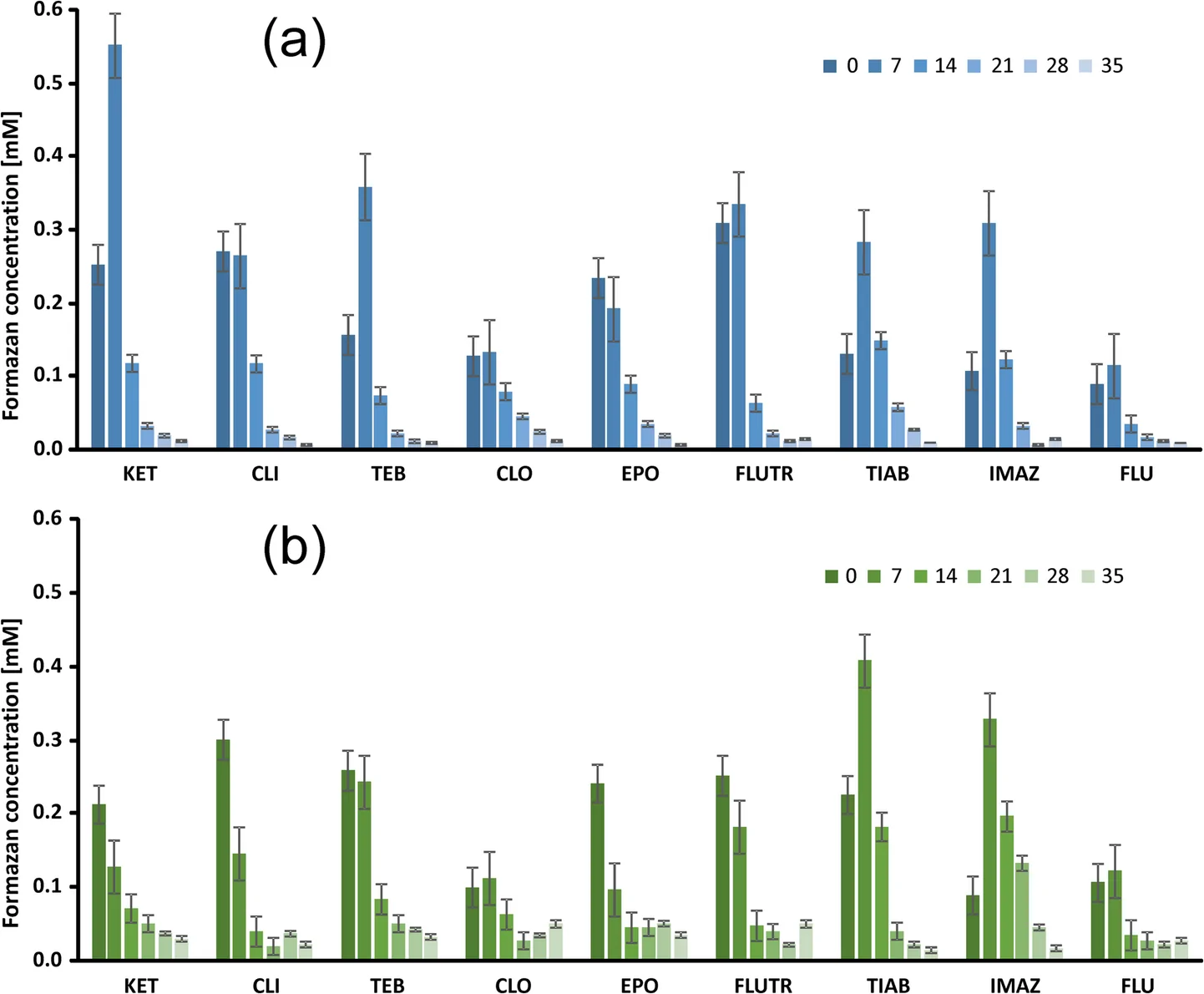
The metabolic activity of microorganisms tested during the biodegradation tests of azole compounds assessed on days 0, 7, 14, 21, 28, and 35 of the experiment. The bars represent standard deviation

The MTT tests were performed to assess the metabolic activity of microorganisms during the biodegradation experiments. The results of these tests conducted on samples containing activated sludge collected during the azoles biodegradation experiments are presented in Fig. 5. In the case of activated sludge from both WWTPs, the increased levels of metabolic activity of microorganisms during the first two weeks correspond with a higher rate of biodegradation and are followed by a significant decrease after three weeks for all compounds. The initial activity (day 0) was in a similar range for both WWTPs, however, in some cases, such as TEB, KET, TIAB, IMAZ at WWTP B, and TIAB, IMAZ at WWTP C, remarkably higher activity of bacteria was observed after seven days of the experiment. This can be influenced by various factors, such as high availability of nutrients at an early stage of the process that stimulate bacterial metabolism. However, the decrease in the final stage may indicate a lack of nutrients and/or the formation of degradation products that exhibit a negative effect on bacteria (although no increase in toxicity was predicted for fish, daphnid and algae with regard to KET, CLI, TEBU, and FLUTR as presented in Table S7).

Similar results in MTT tests were obtained during the biodegradation of bisphenols and tetracyclines with activated sludge inocula. In these biodegradation tests for most contaminants, the bacterial activity was the greatest in the beginning or after 1 week of testing, while considerably lower activity was observed after 2, 3, and 4 weeks [59, 60]. It was particularly evident when biodegradation occurred rapidly and it was shown that the main reason may be the low amount of nutrients, as the decrease of bacterial activity correlated with the lowering of total organic carbon available as the food source [60]. This effect has also been observed in the present study (Figure S14). However, a significant decrease in TOC does not always correlate with biodegradation observed in the test (e.g., for fluconazole). This may be due to the fact that the initial TOC value does not necessarily reflect only the content of the tested compound, but also other compounds introduced together with the activated sludge (even though it was purified). The effect is characteristic for the batch experiments, but under real conditions present in WWTPs, a reduction in microbial activity should therefore not occur, as contaminants enter them continuously. On the other hand, the results of the MTT tests also show that if we would like to isolate bacteria that degrade azoles (or other compounds, if tested), the best time point would probably be one week after the start of the test. However, this requires bacterial colonies containing species that can also cope with these contaminants.

## Conclusions

Based on the results obtained, it can be seen that azoles can be divided into three groups: readily biodegradable (KET, CLI and to a lesser extent EPO), removed primarily via adsorption (CLO and TIAB), and those that are recalcitrant to treatment (FLU and IMAZ). TEB and FLUTR can be classified between the first two groups and their incomplete biodegradation is accompanied by noticeable adsorption. Importantly, CLO and TIAB, although removable by adsorption on activated sludge, may in fact end up in the environment as a result of land application of biosolids. Also, a large part of the identified azole biodegradation products are molecules that have undergone only minor changes, and of similar chronic toxicity to aquatic organisms. Importantly, the two least biodegradable azoles, FLU and IMAZ, are in widespread use. FLU is a widely used medication for fungal infections, and IMAZ is widely used after harvest to protect citrus fruits from mold. For this reason, further research into the removal of azoles should focus on these two compounds, which may pose the greatest threat to living organisms.

## Supplementary Information

Below is the link to the electronic supplementary material.

Supplementary Material 1

## Data Availability

Data will be made available on request.
