# Supplementary material for: Understanding Azole Removal: Contribution of Biodegradation and Bioadsorption
Source: Appl Biochem Biotechnol. 2026 May 22;198(9):6452–69. doi: 10.1007/s12010-026-05741-7 (PMC13570825; doi:10.1007/s12010-026-05741-7)
Supplement: Supplementary file 1 [file 12010_2026_5741_MOESM1_ESM.pdf]

Understanding Azole Removal: Contribution of Biodegradation and Bioadsorption

Julia Płatkiewicz[1]*, Robert Frankowski[1], Tomasz Grześkowiak[1], Alina Pruss[2], Agnieszka Zgoła-Grześkowiak[1]*

[1]Institute of Chemistry and Technical Electrochemistry, Poznan University of Technology, Berdychowo 4, 60-965 Poznań, Poland

[2]Institute of Environmental Engineering and Building Installations, Poznan University of Technology, Berdychowo 4, 60-965 Poznań, Poland

Supplement

* corresponding authors:

AZG tel. +48 616652033, e-mail: agnieszka.zgola-grzeskowiak@put.poznan.pl

JP tel. +48 616653344, e-mail: julia.platkiewicz@doctorate.put.poznan.pl

Table S1. Structures and properties of azole compounds determined in the study

Abbreviation	Chemical name	Chemical structure	CAS	pK_a	$logK_{ow}$	Reference
KET	Ketoconazole	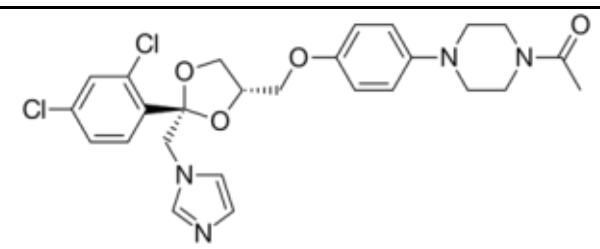	65277-42-1	6.88	4.3	[1]
CLI	Climbazole	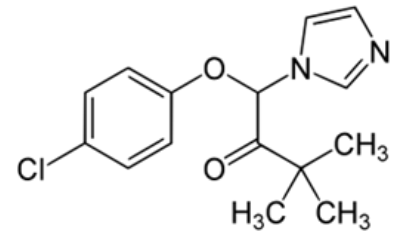	38083-17-9	7.50	3.8	[2]
TEB	Tebuconazole	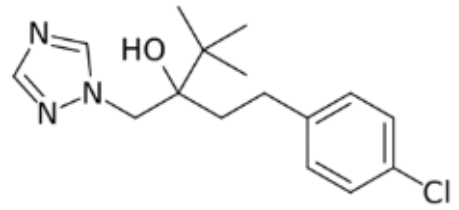	107534-96-3	5.03	3.7	[3,4]
CLO	Clotrimazole	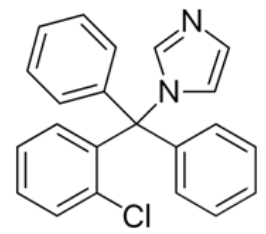	23593-75-1	6.12	4.1	[1]
EPO	Epoxiconazole	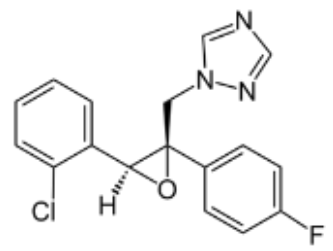	133855-98-8	No data	3.3	[5]
FLUTR	Flutriafol	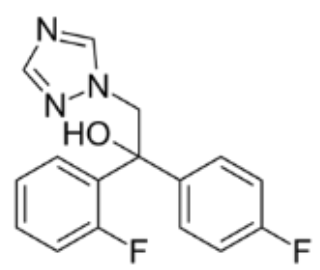	76674-21-0	2.30	2.3	[5]
TIAB	Tiabendazole	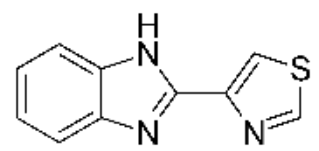	148-79-8	4.7	2.4	[5]

IMAZ	Imazalil	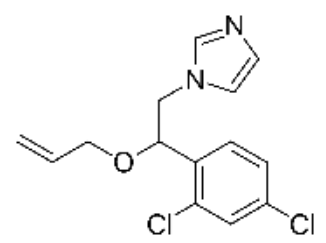	35554-44-0	6.5	3.8	[5]
FLU	Fluconazole	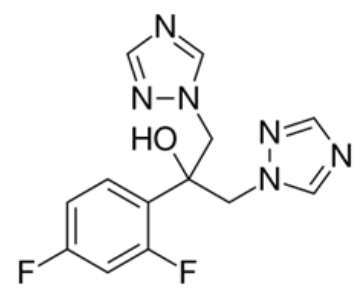	86386-73-4	2.56-2.94 and 11.01	0.4	[1,6]

Table S2. Composition of mineral medium used in biodegradation tests

Compound	Concentration [mg L^{-1}]
$Na_2HPO_3 \cdot 2\ H_2O$	2270
KH_2PO_4	950
NH_4Cl	550
$Na_2EDTA \cdot 2\ H_2O$	12.74
$ZnSO_4 \cdot 7\ H_2O$	2
$CaCl_2 \cdot 2\ H_2O$	1
$FeSO_4 \cdot 7\ H_2O$	5
$(NH_4)_6Mo_7O_{24}$	0.2
$CuSO_4 \cdot 5\ H_2O$	0.2
$CoCl_2$	0.2
$MnSO_4 \cdot H_2O$	1.04
$MgSO_4 \cdot 7\ H_2O$	120

Table S3. Parameters of mass spectrometric detection characteristic of the particular analytes

Azole	Retention time [min]	DP[a] [V]	Multiple reaction monitoring transitions (primary ion $[M+H]^+$ m/z → fragment ion m/z)							
			Analytical	EP[b] [V]	CE[c] [eV]	CXP[d] [V]	Confirmatory	EP[b] [V]	CE[c] [eV]	CXP[d] [V]
KET	3.2	30	531 → 82	2	60	3	531 → 135	6	44	10
CLI	3.5	50	293 → 197	5	24	12	293 → 69	7	32	2
TEB	5.8	30	308 → 70	5	37	12	308 → 125	4	44	6
CLO	3.7	60	277 → 165	10	29	10	277 → 242	10	28	10
EPO	5.7	50	330 → 121	3	28	6	330 → 91	3	62	4
FLUTR	4.5	30	302 → 70	3	25	7	302 → 123	4	37	6
TIAB	1.3	10	202 → 175	2	30	10	202 → 131	2	43	10
IMAZ	3.1	10	297 → 159	3	31	8	297 → 201	3	26	11
FLU	2.4	30	307 → 238	10	25	5	307 → 220	10	27	10

[a]DP – declustering potential, [b]EP – entrance potential, [c]CE – collision energy, [d]CXP – collision cell exit potential

Table S4. Validation parameters of the developed LC-MS/MS method

Azole	LOD [ng L^{-1}]	LOQ [ng L^{-1}]	Linearity [ng L^{-1}]		R^2
			min	max	
KET	64.3	214.3	4000.0	$2 \cdot 10^5$	0.9998
CLI	15.4	51.2	1000.0	$1 \cdot 10^5$	0.9999
TEB	20.0	66.7	2000.0	$1 \cdot 10^6$	0.9995
CLO	10.1	33.8	500.0	$5 \cdot 10^5$	0.9999
EPO	4.6	15.3	500.0	$5 \cdot 10^5$	0.9997
FLUTR	11.3	37.7	500.0	$2.5 \cdot 10^5$	0.9993
TIAB	43.7	145.8	1000.0	$2.5 \cdot 10^5$	0.9995
IMAZ	36.0	120.0	100.0	$1 \cdot 10^5$	0.9998
FLU	17.7	59.2	1000.0	$5 \cdot 10^5$	0.9999

Table S5. Recovery and precision (relative standard deviation) for the determination of azoles adsorbed on the activated sludge

Azole	Recovery [%]	Precision [%]
KET	85.9	8.8
CLI	86.7	3.9
TEB	94.3	5.7
CLO	79.0	15.9
EPO	98.7	1.2
FLUTR	91.7	2.5
TIAB	77.2	3.8
IMAZ	93.7	2.4
FLU	100.6	3.0

Table S6. Results from ANOVA and Tukey tests verifying statistical difference between the mean removal of azoles in the biodegradation tests with inocula from rural WWTP and urban WWTP. The difference is confirmed only for p-values lower than 0.05 in ANOVA test and difference between mean values greater than Tukey critical value (alpha = 0.05)

Azole	p-value	Tukey critical value	Difference between mean values
KET	0.03	0.747	0.875
CLI	0.01	6.12	10.03
TEB	0.03	9.52	11.21
CLO	0.001	10.43	31.04
EPO	0.002	13.84	36.72
FLUTR	0.01	8.17	15.16
TIAB	0.01	3.10	5.59
IMAZ	0.33	5.07	2.04
FLU	0.16	21.11	3.03

Table S7. Comparison of chronic toxicity (ChV) of azoles and their biodegradation products

Compound	Structure	Organism	ChV concentration, mg/L
Ketoconazole	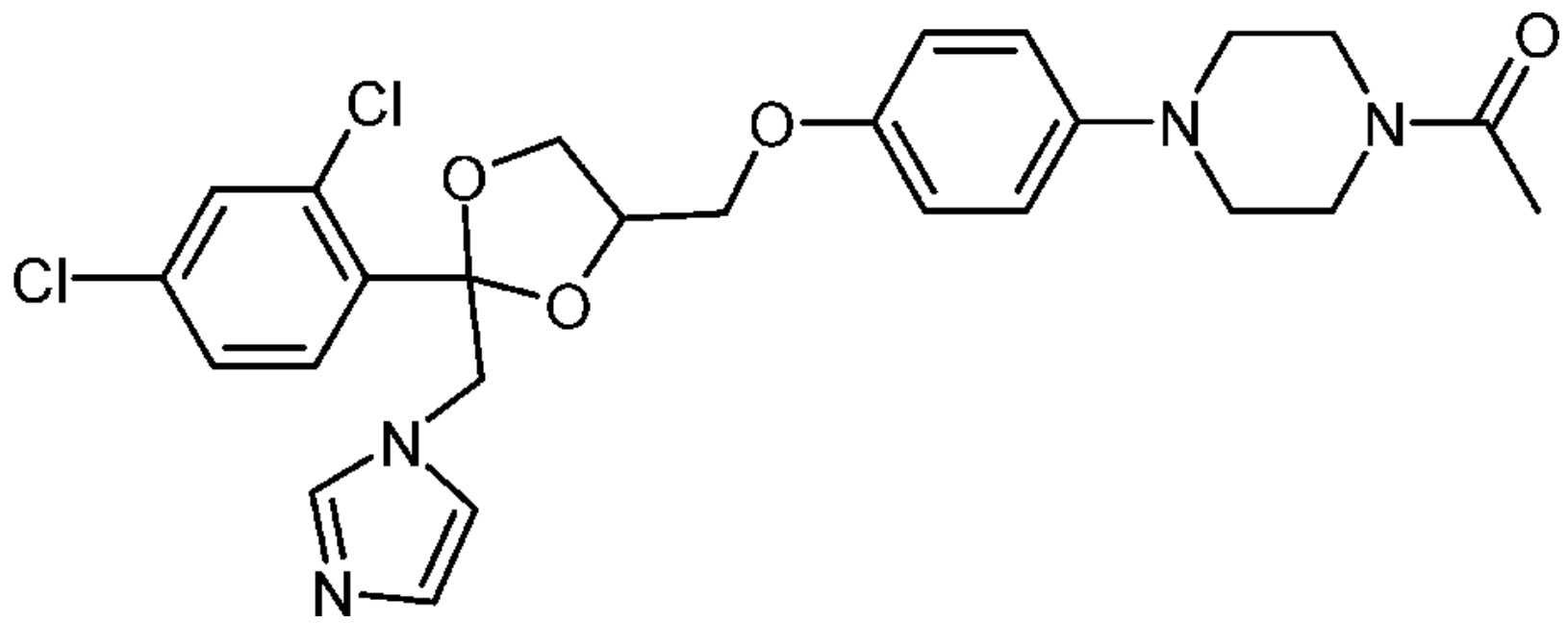	Fish<br>Daphnid<br>Green algae	0.031<br>0.020<br>0.114
Ketoconazole<br>degradation product m/z 343	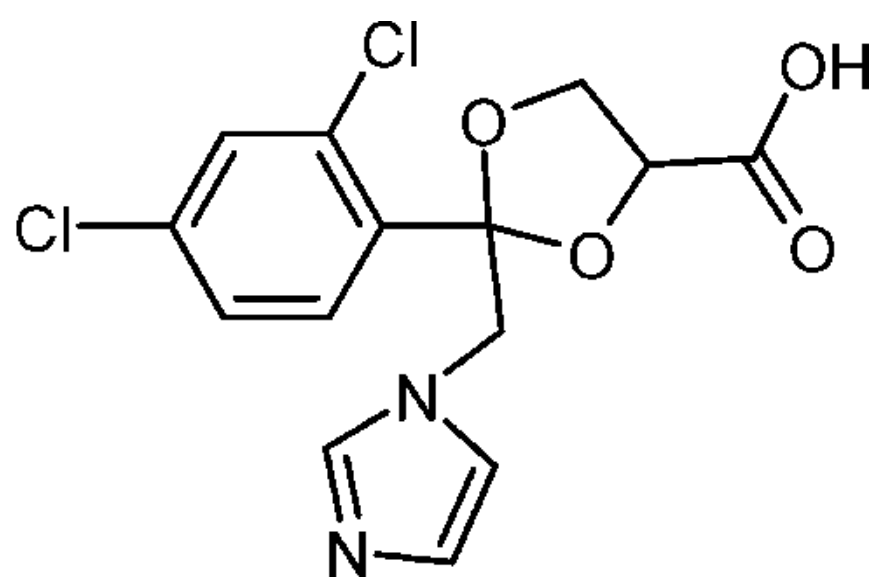	Fish<br>Daphnid<br>Green algae	0.412<br>0.354<br>1.47
Ketoconazole<br>degradation product m/z 329	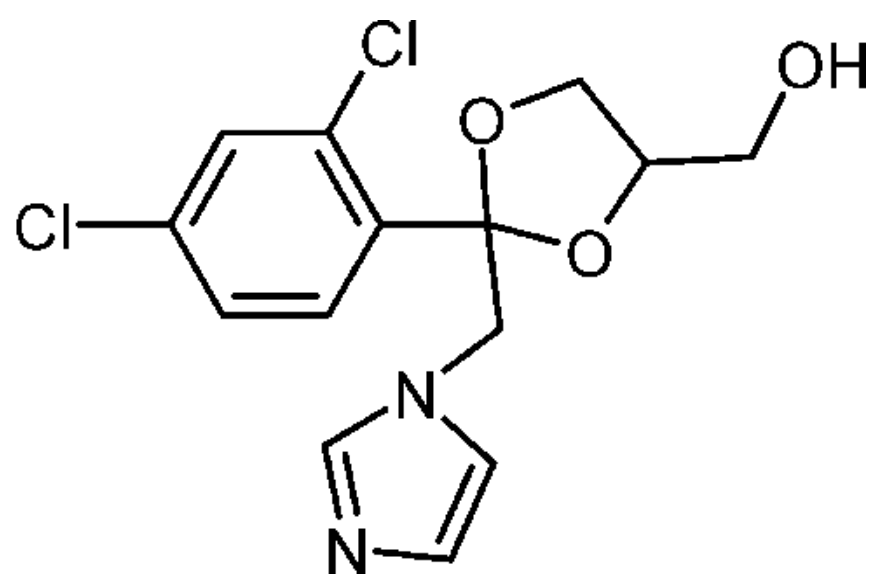	Fish<br>Daphnid<br>Green algae	0.058<br>0.057<br>0.203

Ketoconazole degradation product m/z 315	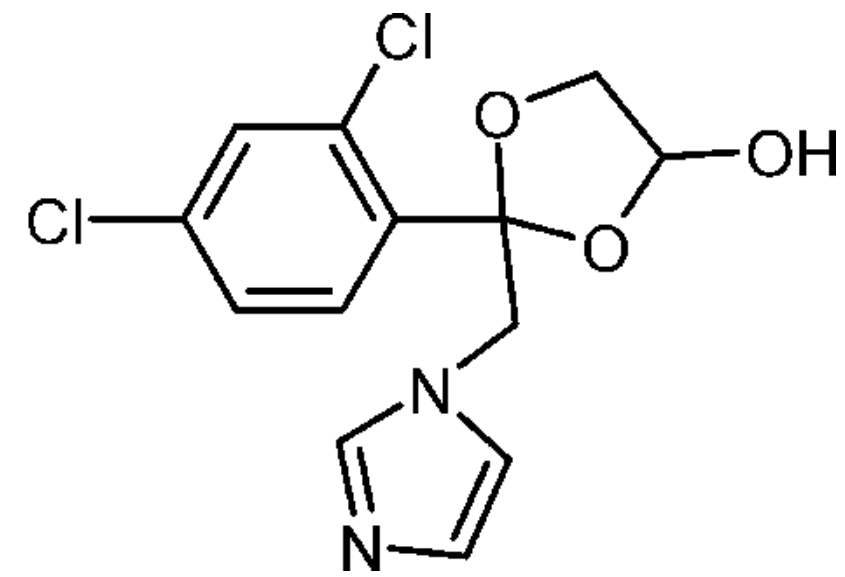	Fish	0.221
		Daphnid	0.351
		Green algae	0.711
Ketoconazole degradation product m/z 235	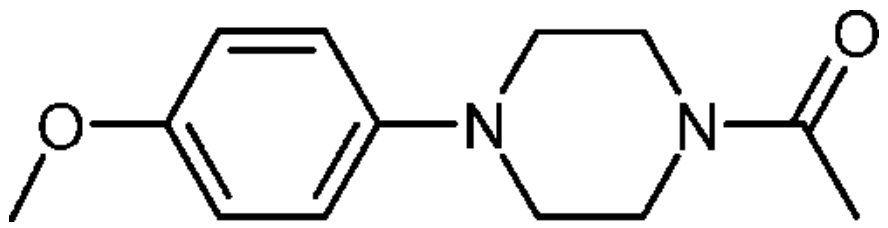	Fish	2.17
		Daphnid	31.2
		Green algae	10.0
Cllimbazole	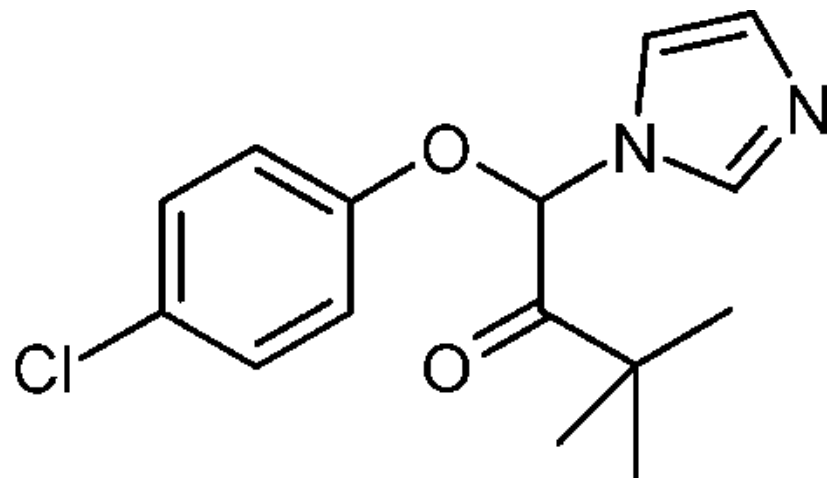	Fish	0.034
		Daphnid	0.029
		Green algae	0.121
Climbazole degradation product m/z 275	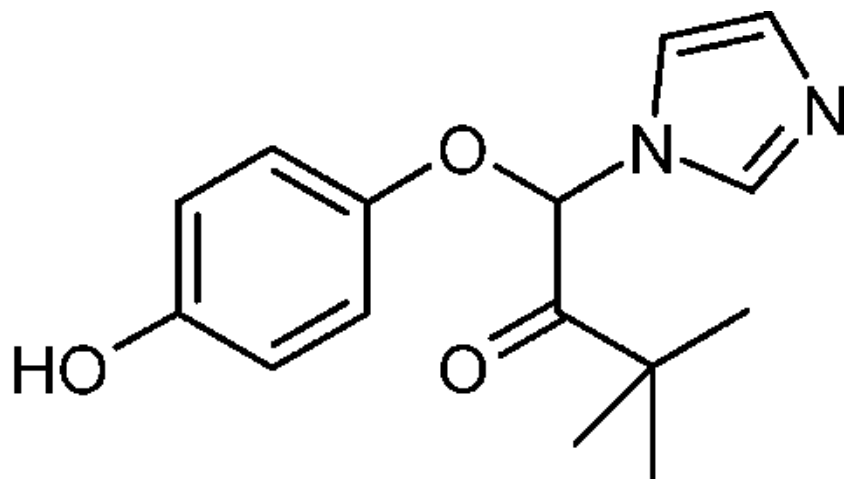	Fish	0.101
		Daphnid	0.128
		Green algae	0.337

Cllimbazole<br>degradation product m/z 295	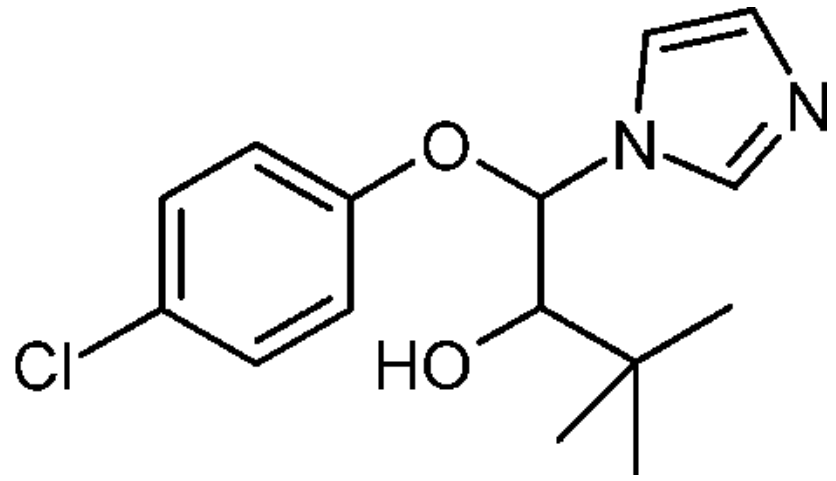	Fish<br>Daphnid<br>Green algae	0.034<br>0.029<br>0.121
Cllimbazole<br>degradation product m/z 253	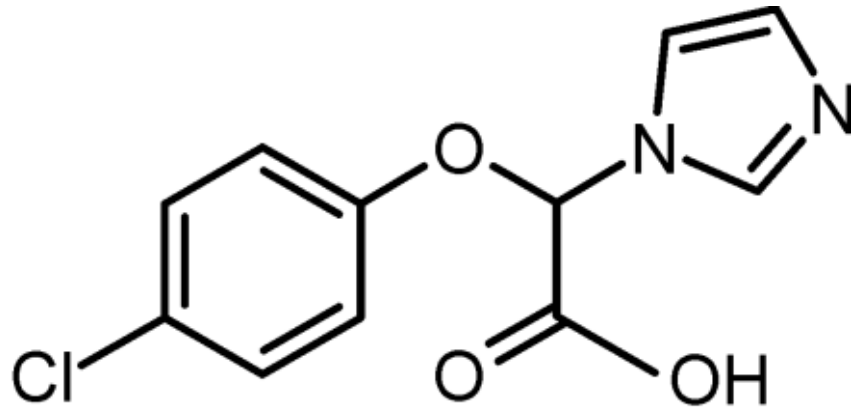	Fish<br>Daphnid<br>Green algae	1.43<br>2.09<br>4.64
Tebuconazole	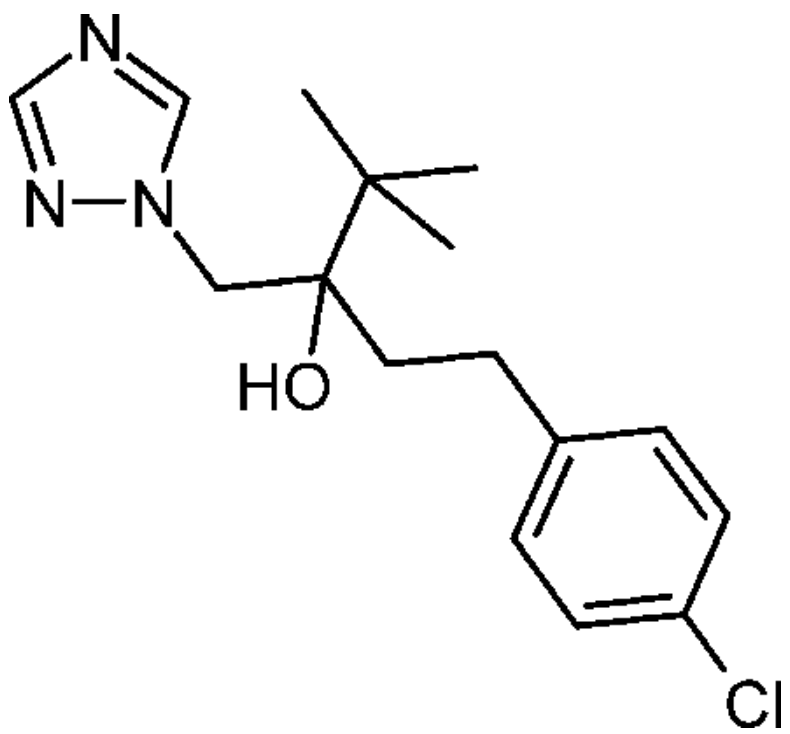	Fish<br>Daphnid<br>Green algae	0.017<br>0.194<br>0.494

Tebuconazole<br>degradation product m/z 324	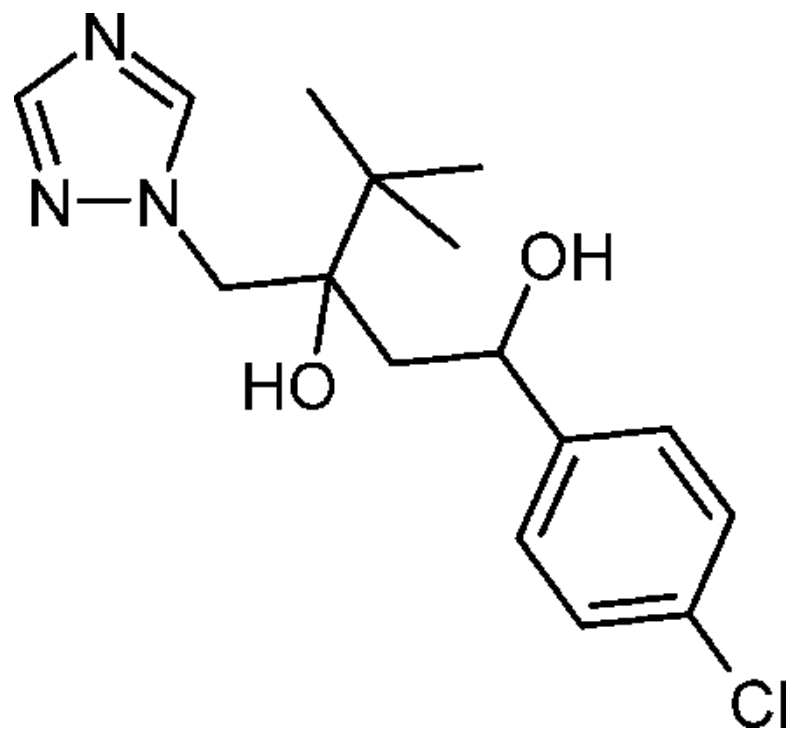	Fish<br>Daphnid<br>Green algae	0.087<br>1.26<br>1.32
Tebuconazole<br>degradation product m/z 324	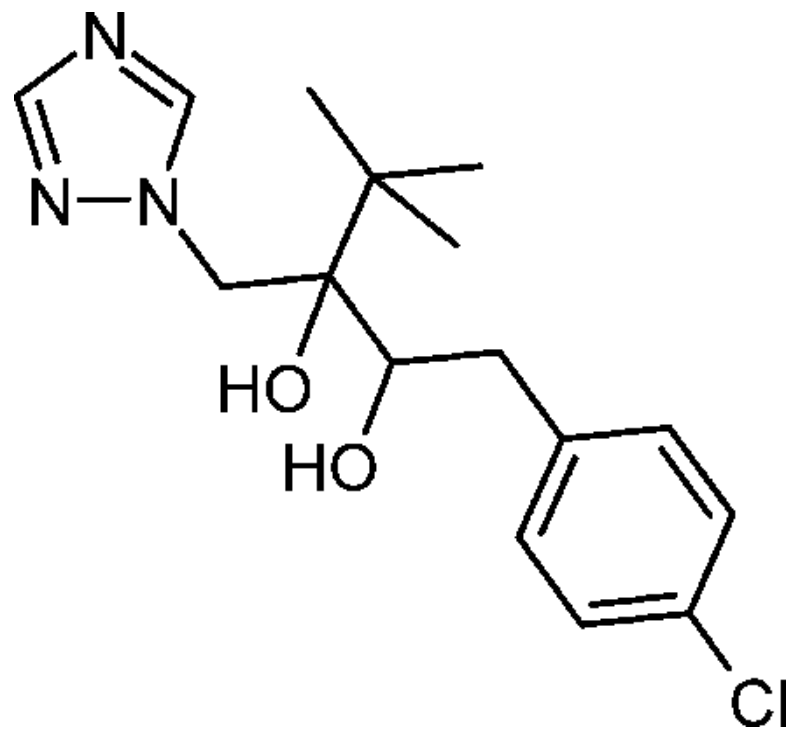	Fish<br>Daphnid<br>Green algae	0.087<br>1.26<br>1.32
Tebuconazole<br>degradation product m/z 324	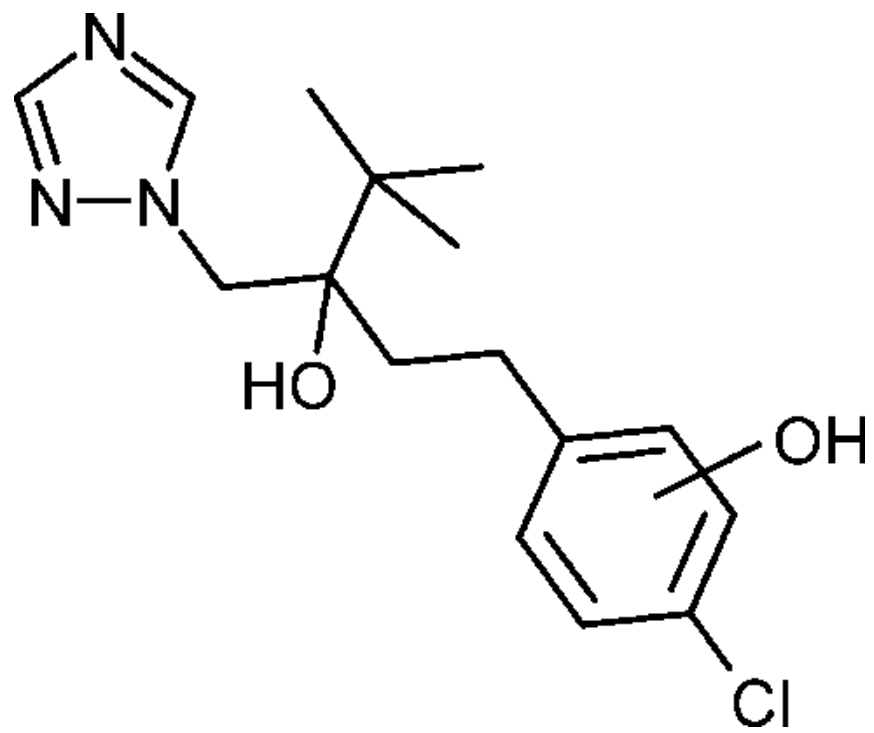	Fish<br>Daphnid<br>Green algae	0.035<br>0.441<br>0.772

Tebuconazole degradation product m/z 274	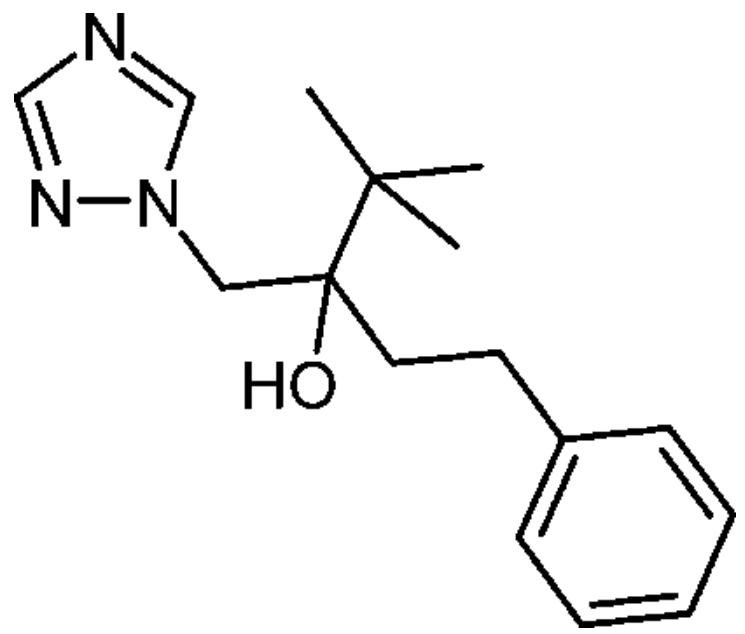	Fish<br>Daphnid<br>Green algae	0.037<br>0.485<br>0.747
Flutriafol	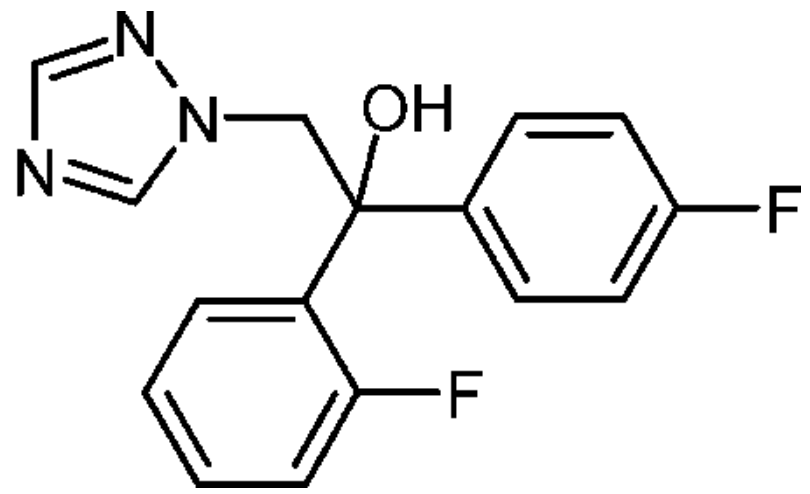	Fish<br>Daphnid<br>Green algae	0.113<br>1.72<br>1.50
Flutriafol degradation product m/z 128	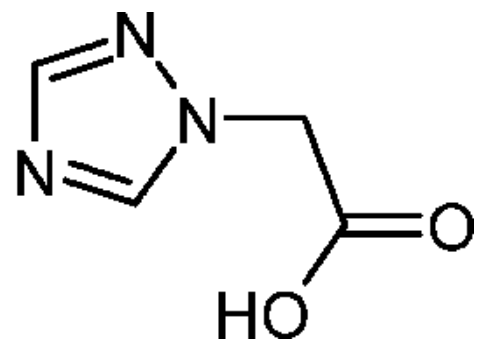	Fish<br>Daphnid<br>Green algae	169<br>$6.45*10^3$<br>209
Flutriafol degradation product m/z 128	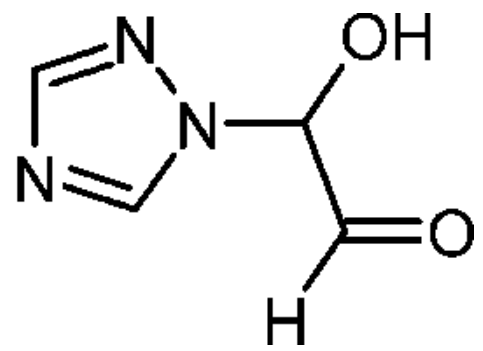	Fish<br>Daphnid<br>Green algae	33.4<br>$1.42*10^3$<br>31.4

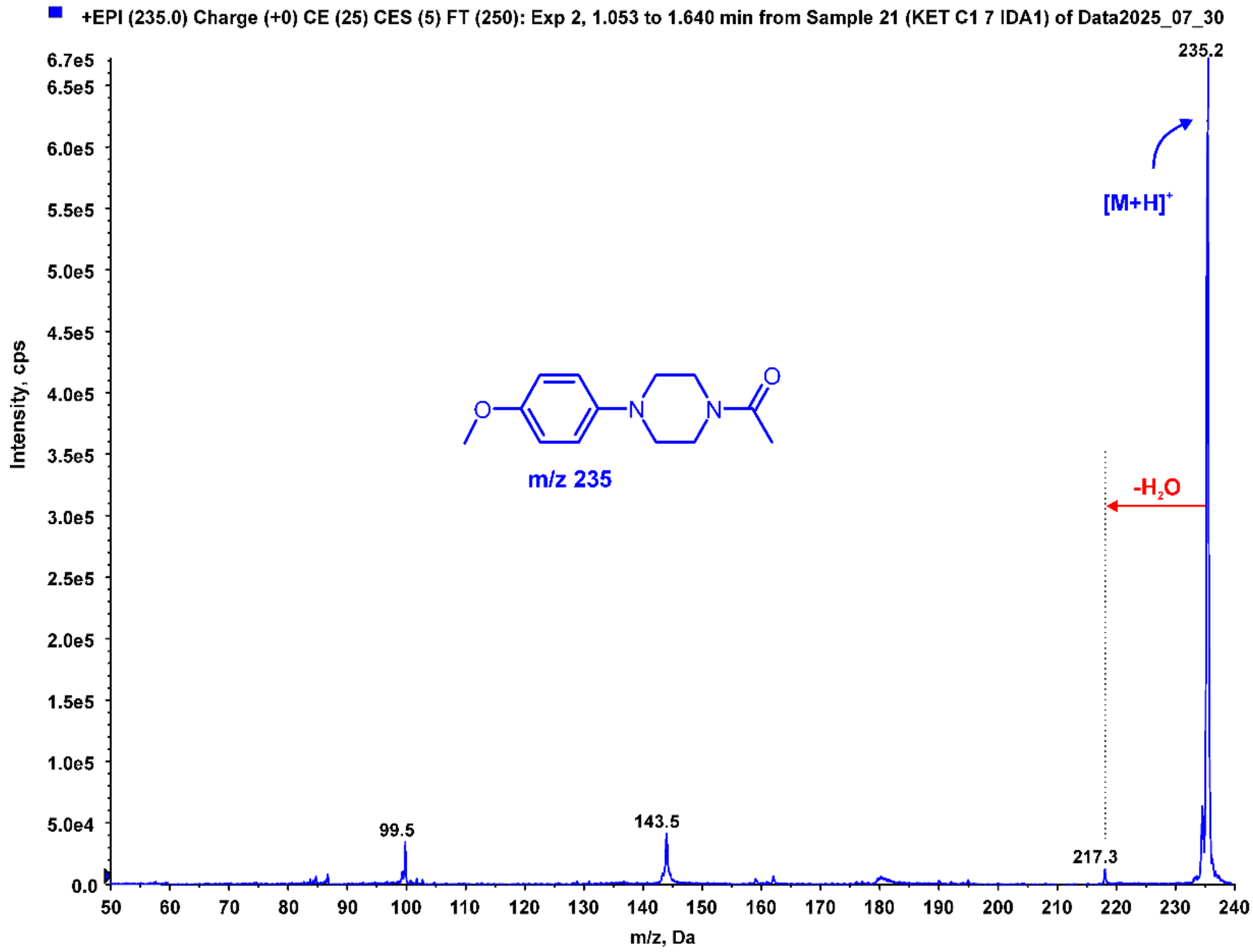

Figure S1. Enhanced product ion mass spectrum of KET degradation product at m/z 235

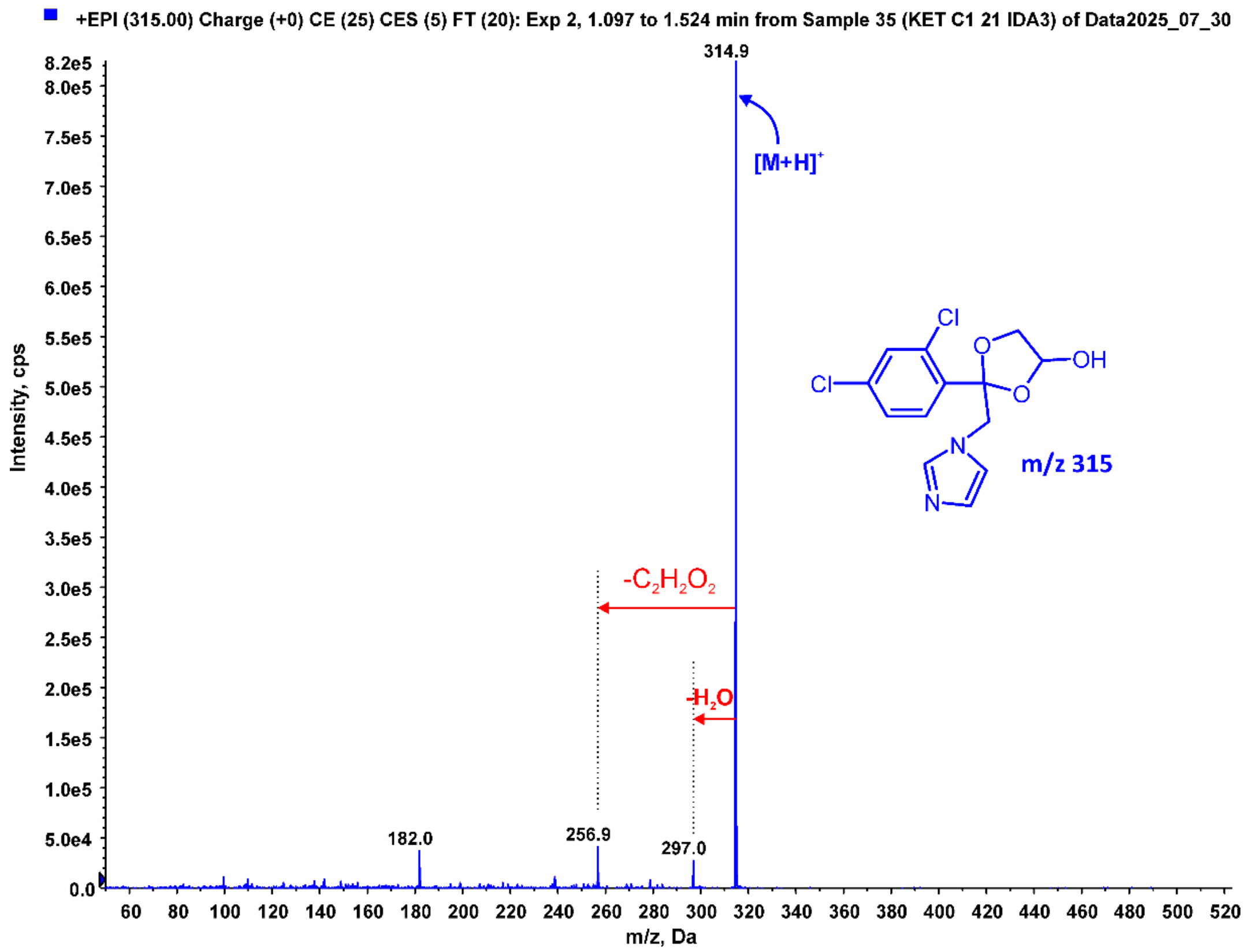

Figure S2. Enhanced product ion mass spectrum of KET degradation product at m/z 315

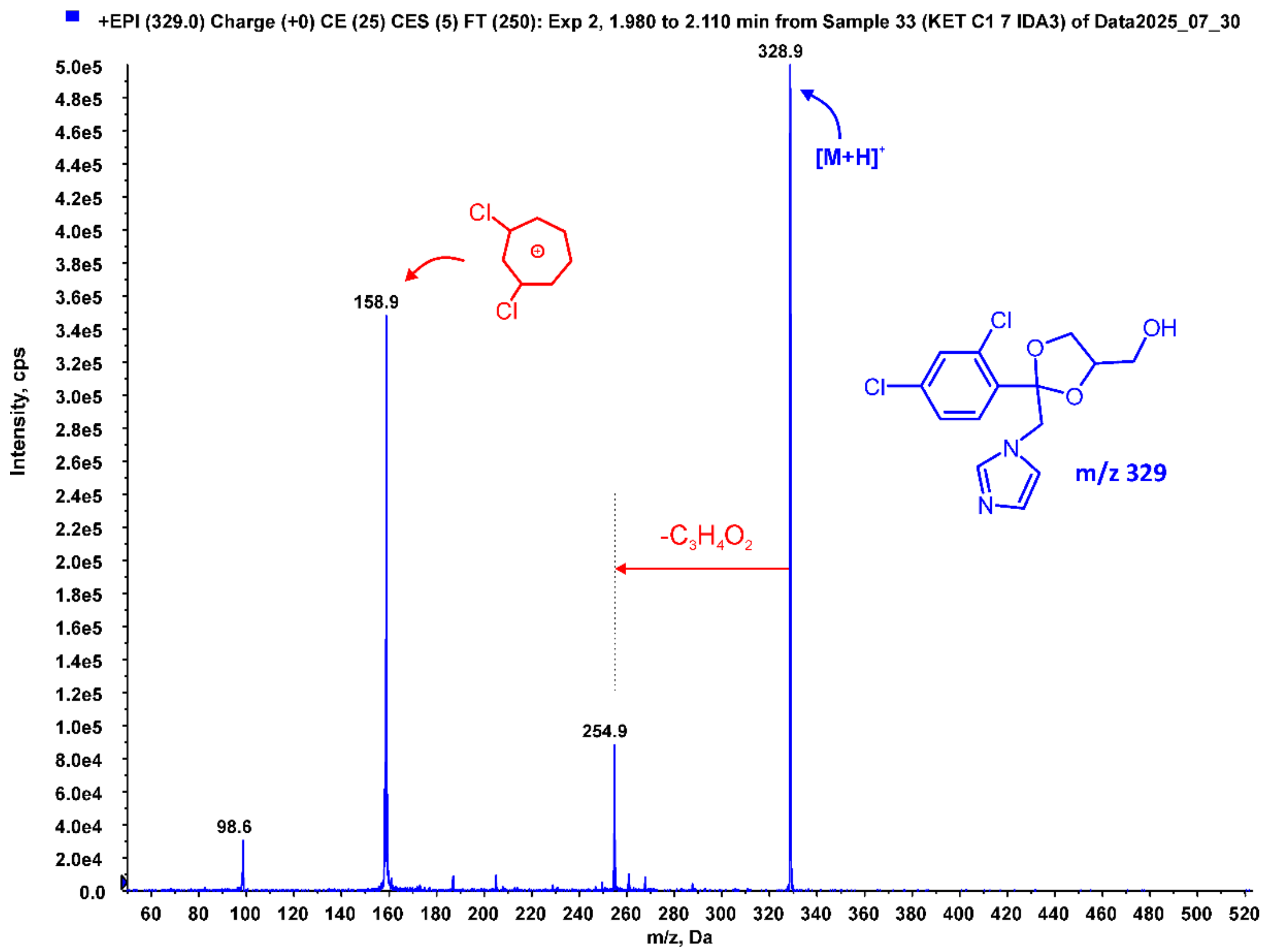

Figure S3. Enhanced product ion mass spectrum of KET degradation product at m/z 329

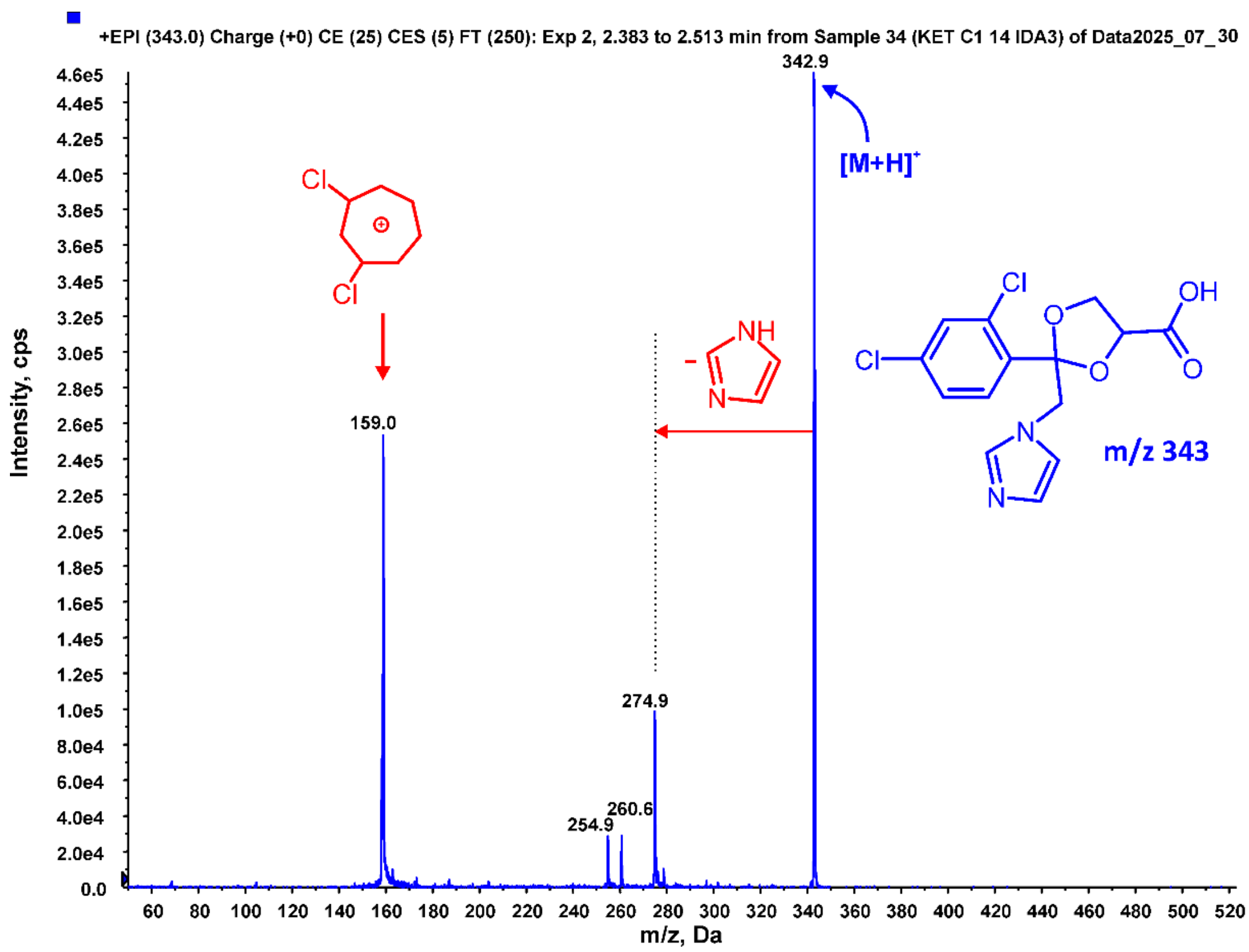

Figure S4. Enhanced product ion mass spectrum of KET degradation product at m/z 343

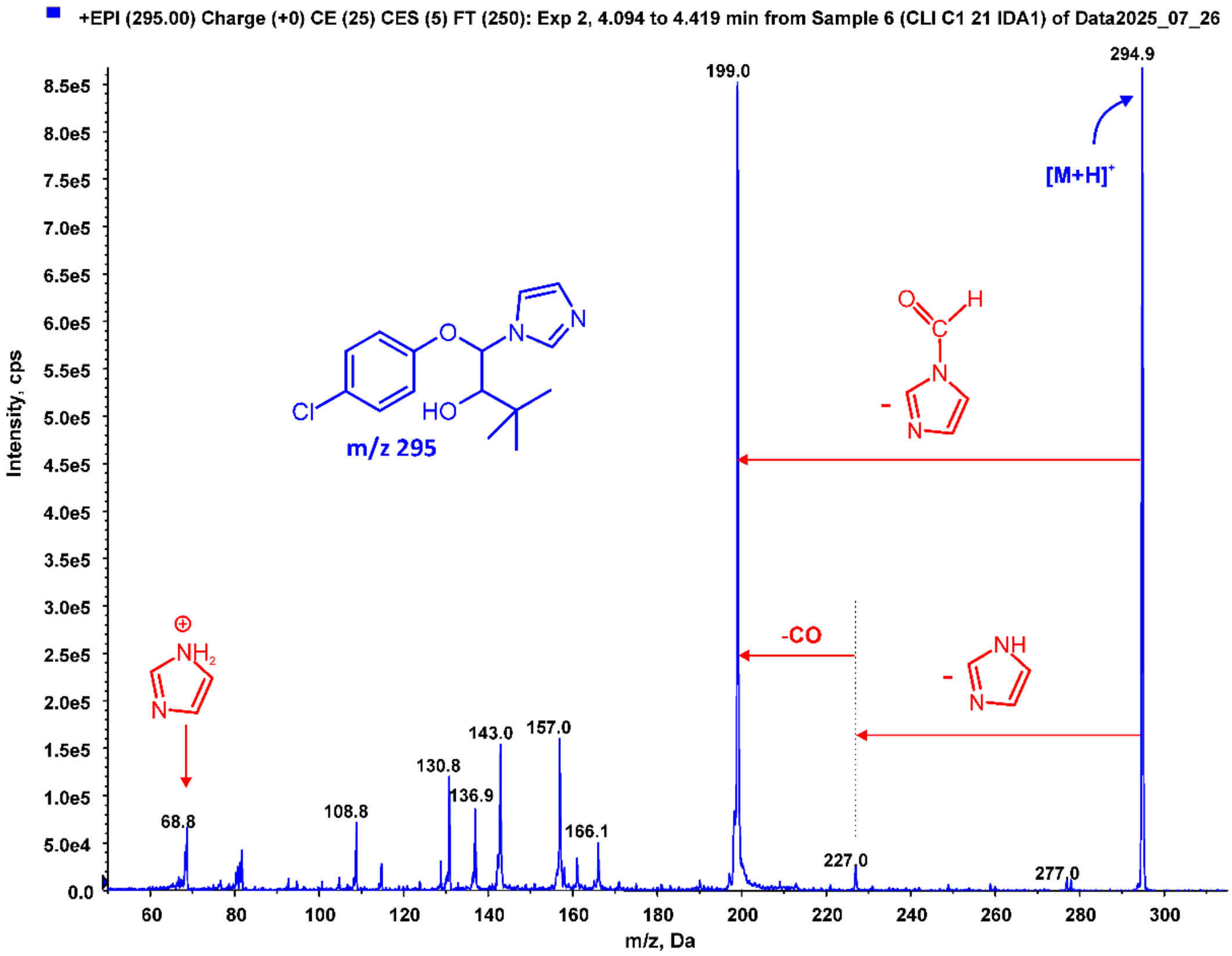

Figure S5. Enhanced product ion mass spectrum of CLI degradation product at m/z 295

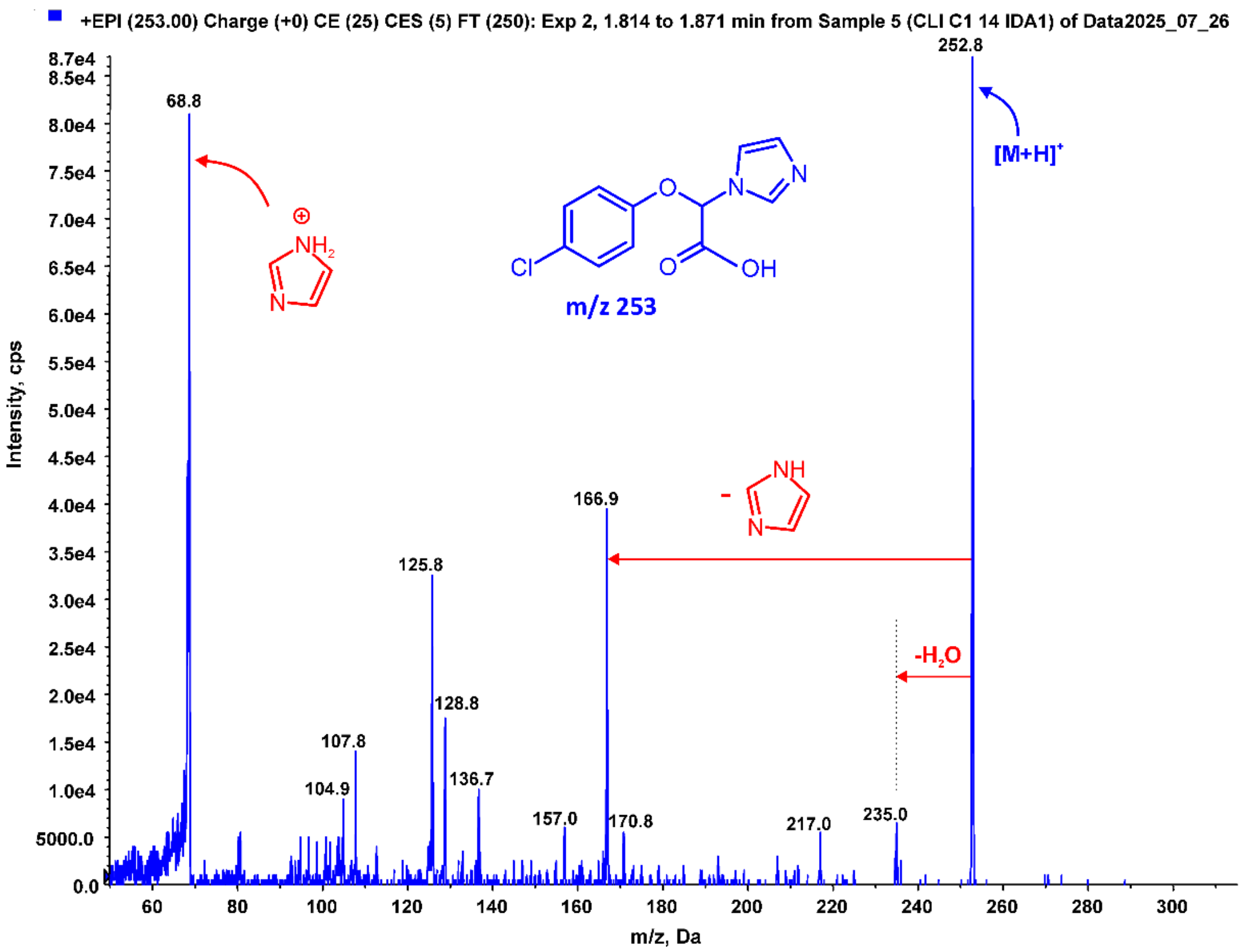

Figure S6. Enhanced product ion mass spectrum of CLI degradation product at m/z 253

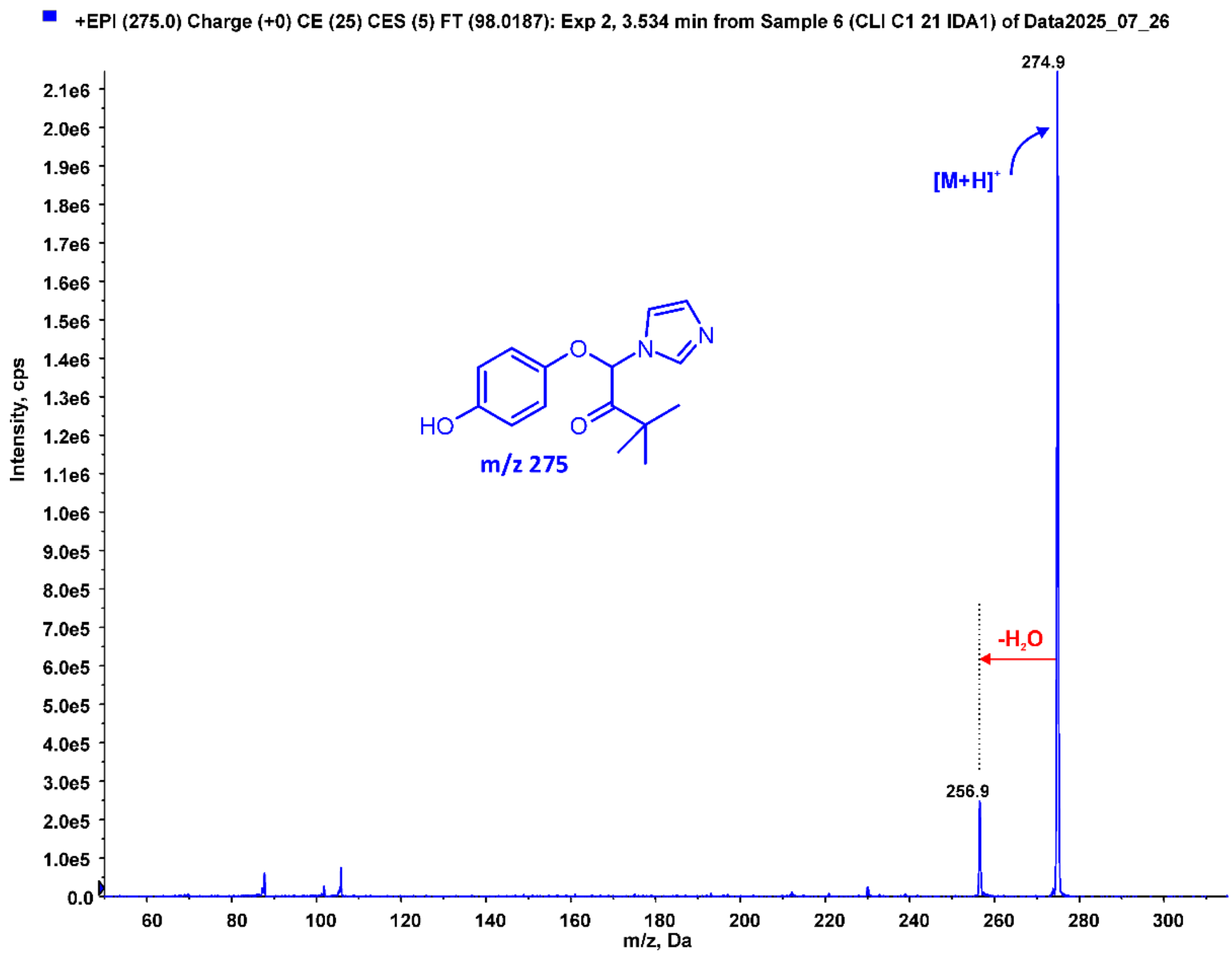

Figure S7. Enhanced product ion mass spectrum of CLI degradation product at m/z 275

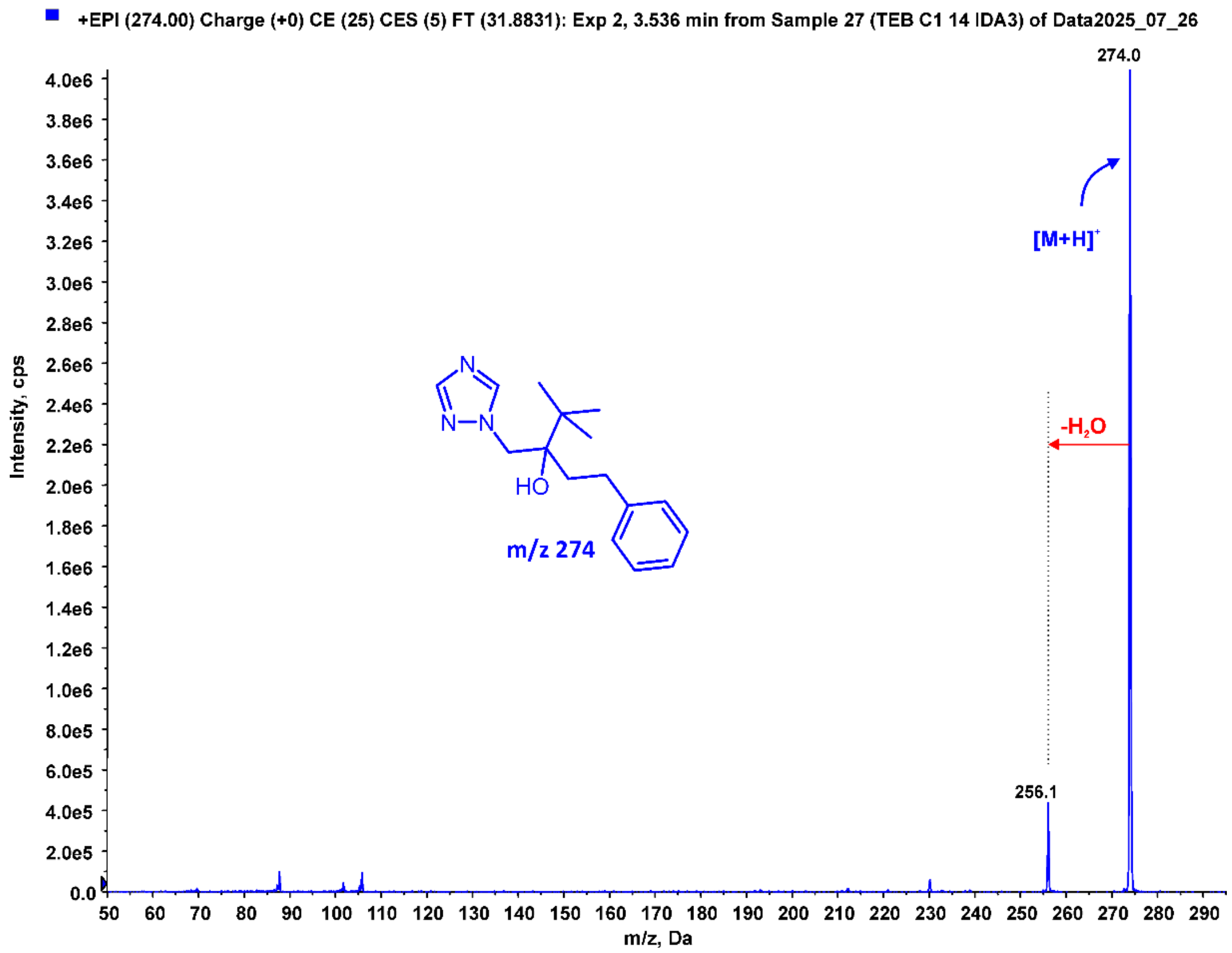

Figure S8. Enhanced product ion mass spectrum of TEB degradation product at m/z 274

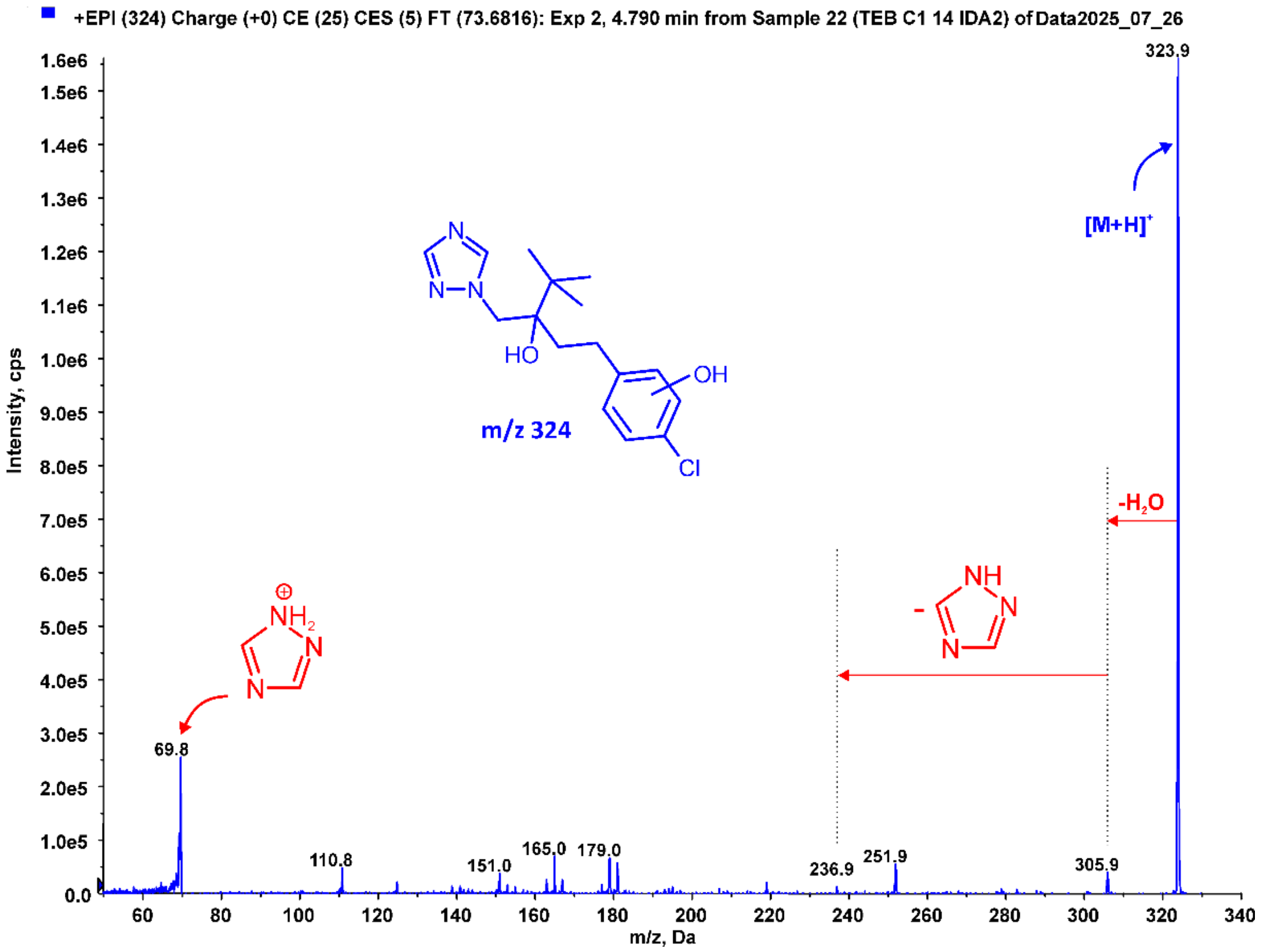

Figure S9. Enhanced product ion mass spectrum of TEB degradation product at m/z 324

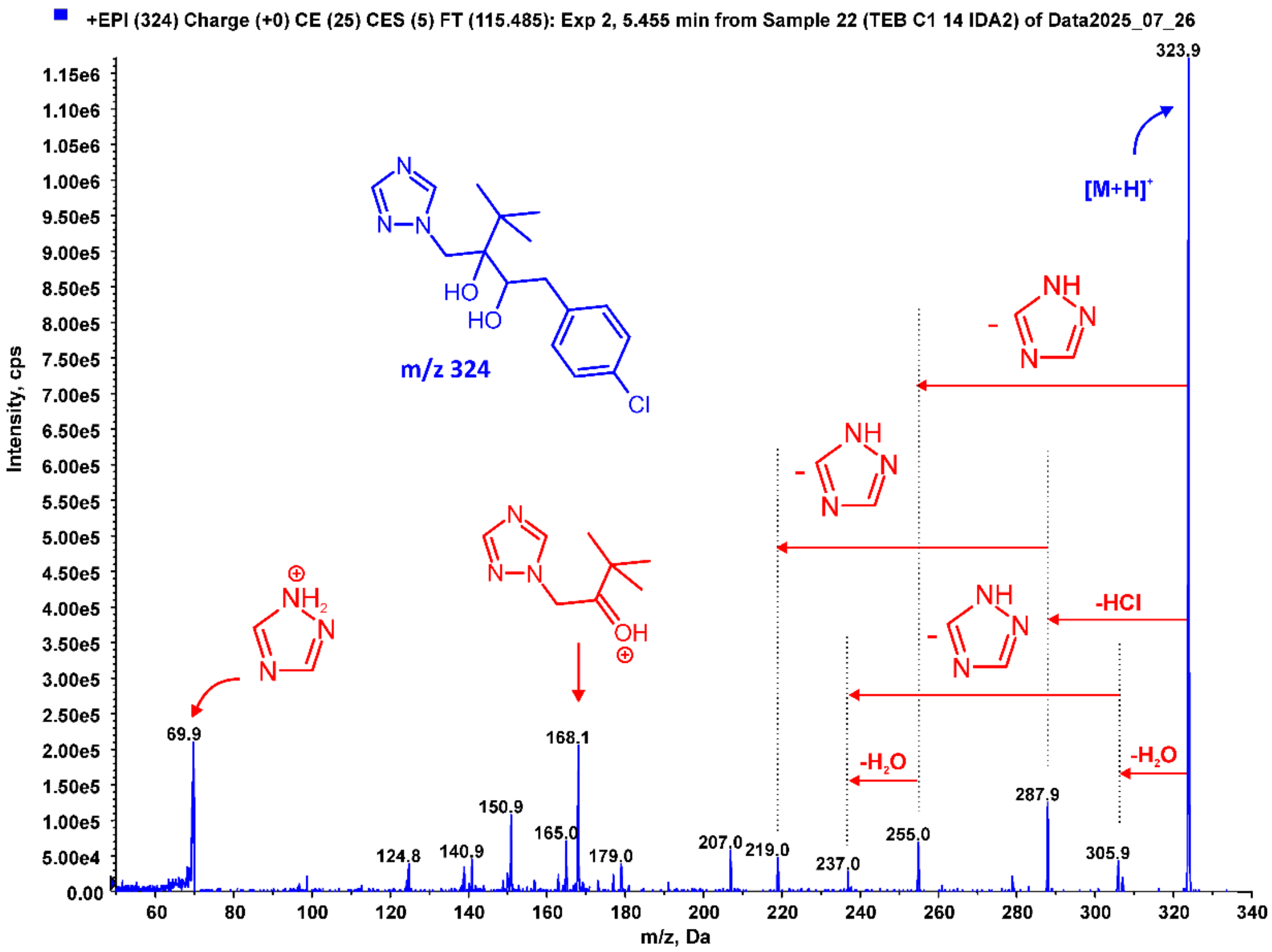

Figure S10. Enhanced product ion mass spectrum of TEB degradation product at m/z 324

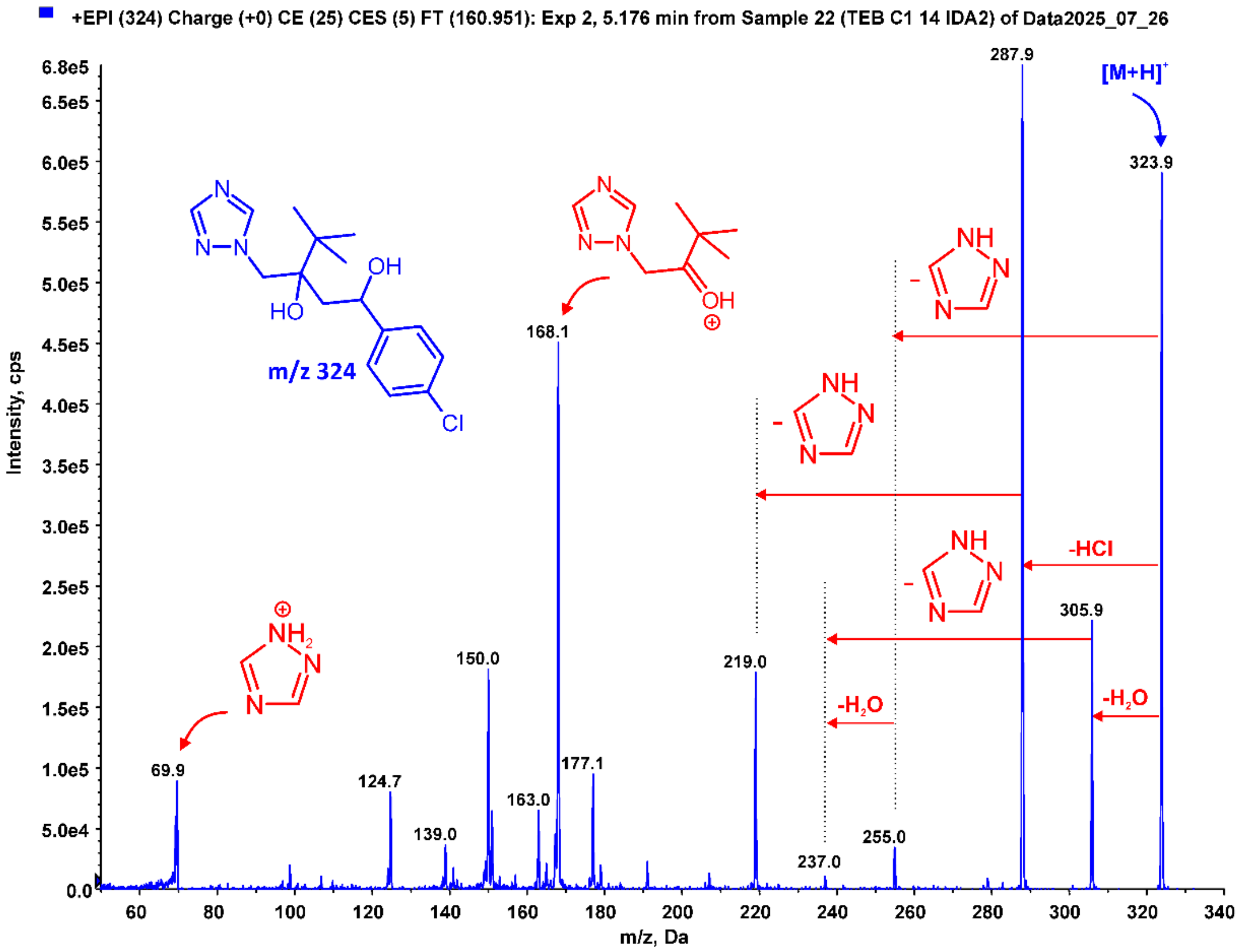

Figure S11. Enhanced product ion mass spectrum of TEB degradation product at m/z 324

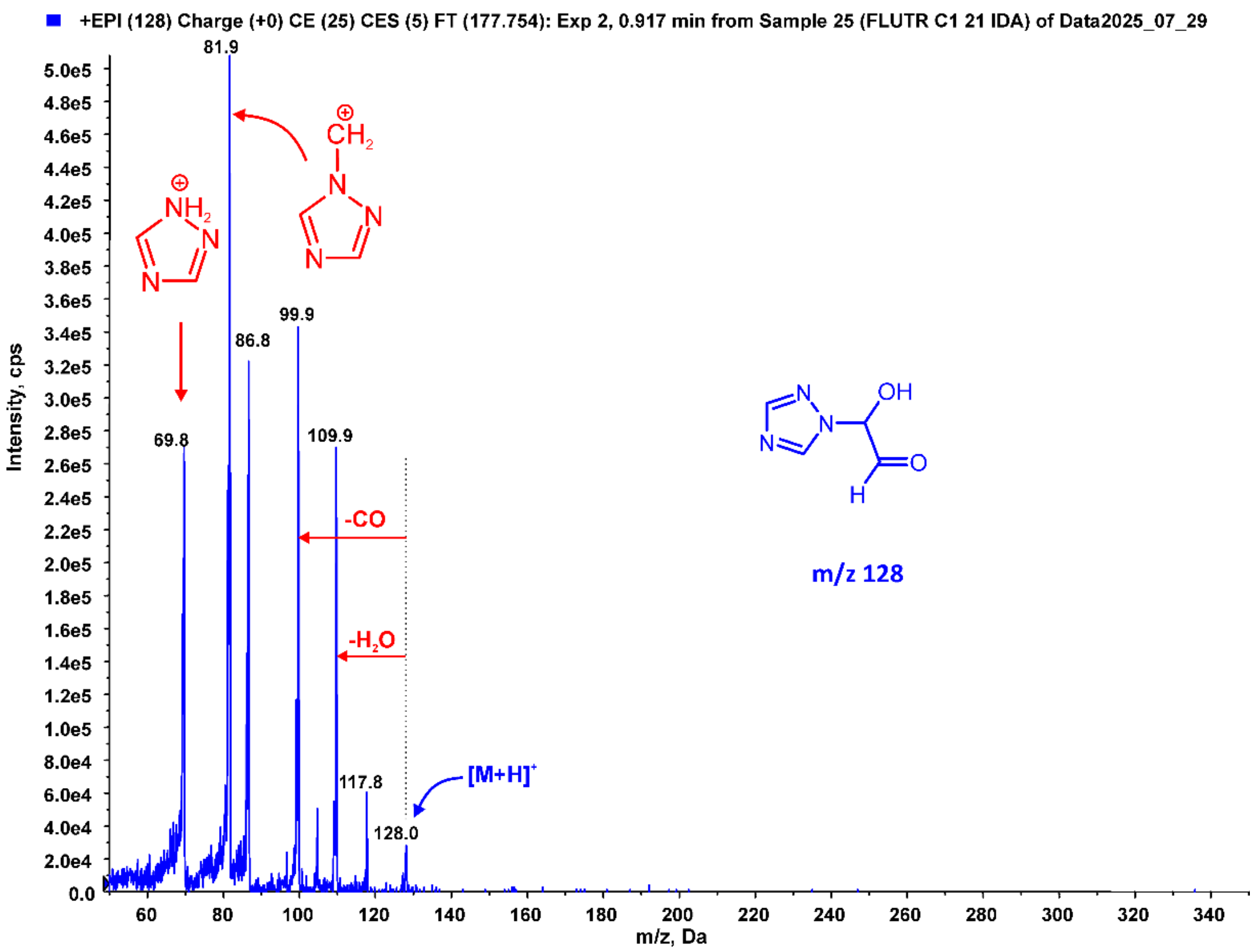

Figure S12. Enhanced product ion mass spectrum of FLUTR degradation product at m/z 128

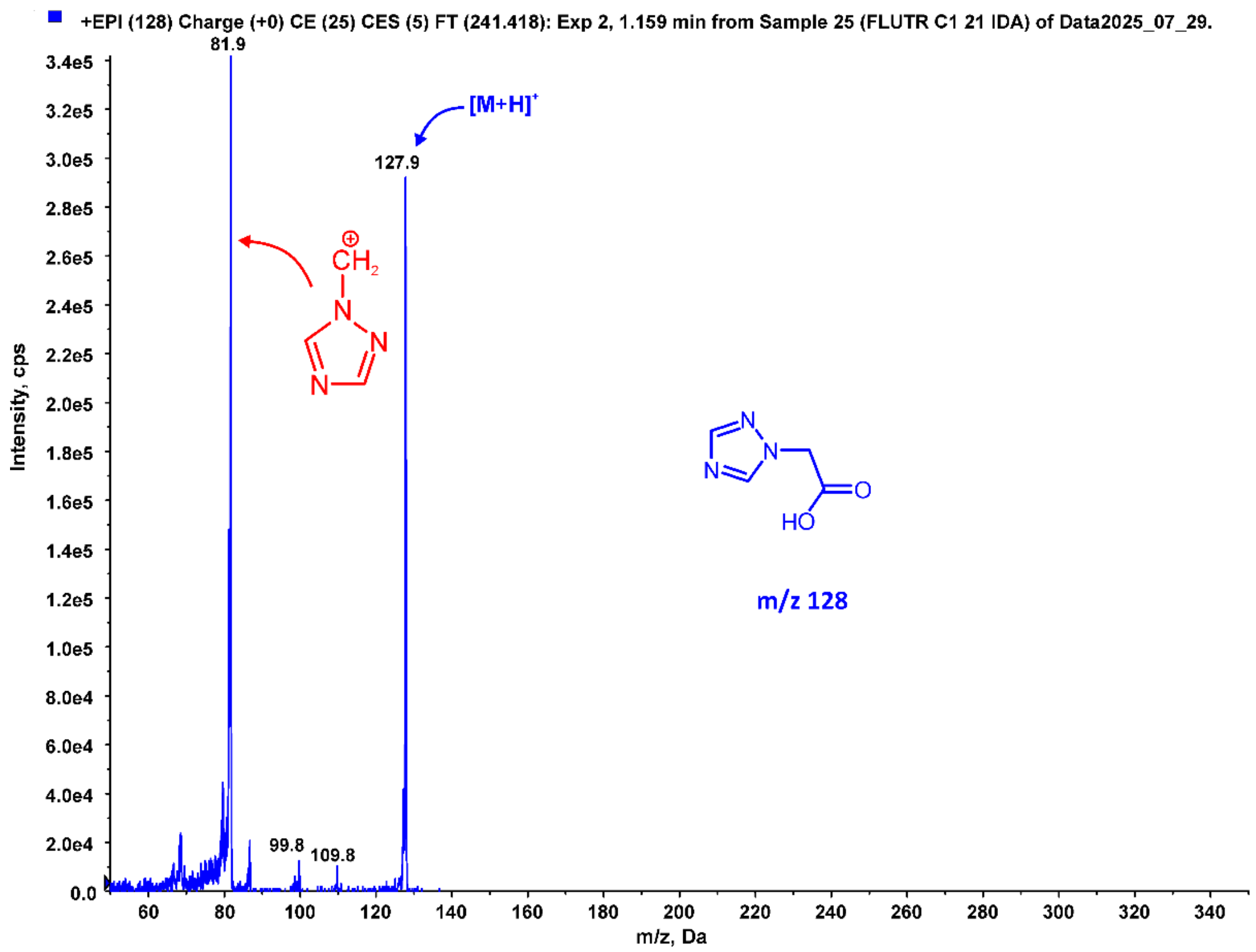

Figure S13. Enhanced product ion mass spectrum of FLUTR degradation product at m/z 128

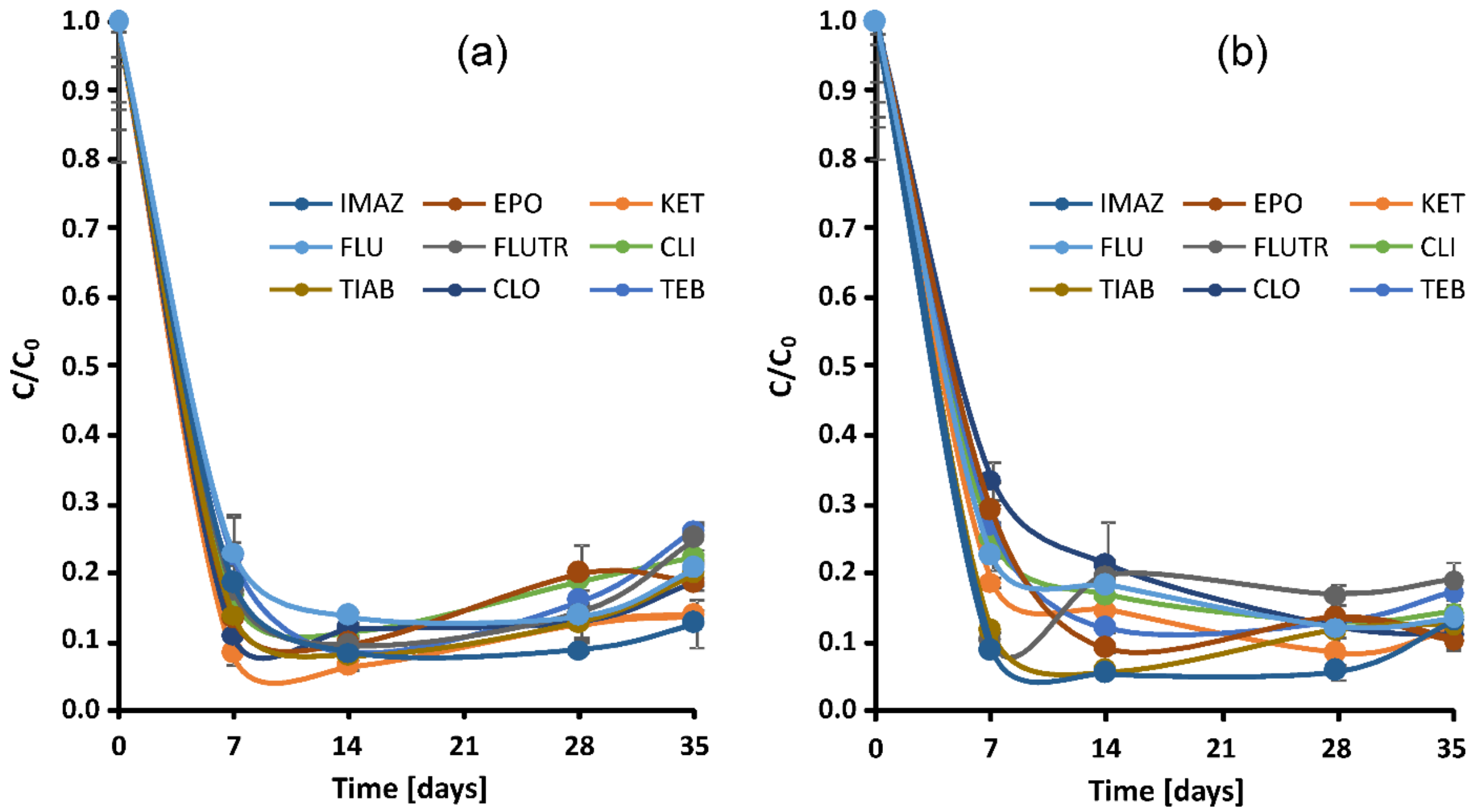

Figure S14. Total organic carbon content measured during the biodegradation tests of azole compounds with activated sludge from (a) rural WWTP and (b) urban WWTP. The bars represent standard deviation

Literature

[1] J. Casado, I. Rodríguez, M. Ramil, R. Cela, Selective determination of antimycotic drugs in environmental water samples by mixed-mode solid-phase extraction and liquid chromatography quadrupole time-of-flight mass spectrometry, J Chromatogr A 1339 (2014) 42–49. https://doi.org/10.1016/j.chroma.2014.02.087.

[2] Z.-F. Chen, G.-G. Ying, Occurrence, fate and ecological risk of five typical azole fungicides as therapeutic and personal care products in the environment: A review, Environ Int 84 (2015) 142–153. https://doi.org/10.1016/j.envint.2015.07.022.

[3] N. Cui, P. Wang, N. Xu, Sorption behaviour of tebuconazole on microplastics: kinetics, isotherms and influencing factors, Environ Technol 44 (2023) 3937–3948. https://doi.org/10.1080/09593330.2022.2077133.

[4] Q. Huang, Y. Yu, C. Tang, X. Peng, Determination of commonly used azole antifungals in various waters and sewage sludge using ultra-high performance liquid chromatography–tandem mass spectrometry, J Chromatogr A 1217 (2010) 3481–3488. https://doi.org/10.1016/j.chroma.2010.03.022.

[5] University of Hertfordshire, 2020. PPDB: Pesticide Properties DataBase, (n.d.). http://sitem.herts.ac.uk/aeru/ppdb/en/index.htm. (accessed November 6, 2024).

[6] H.A. Assress, H. Nyoni, B.B. Mamba, T.A.M. Msagati, Occurrence and risk assessment of azole antifungal drugs in water and wastewater, Ecotoxicol Environ Saf 187 (2020) 109868. https://doi.org/10.1016/j.ecoenv.2019.109868.
